# Pharmacological effects, formulations, and clinical research progress of curcumin

**DOI:** 10.3389/fphar.2025.1509045

**Published:** 2025-03-17

**Authors:** Minghui Hao, Chungang Zhang, Ti Wang, Heng Hu

**Affiliations:** ^1^ College of Pharmacy, Liaoning University of Traditional Chinese Medicine, Dalian, China; ^2^ Department of Pharmacy, Changzhi Medical College, Changzhi, China; ^3^ Qimeng Co., LTD, Chifeng, China

**Keywords:** curcumin, formulation, pharmacological effect, clinical research, review

## Abstract

Curcumin, a polyphenolic compound derived from the traditional Chinese medicine turmeric, which has a variety of pharmacological effects, including anti-cancer, anti-inflammatory, antioxidant, and antiviral properties. However, its clinical application is hindered by low solubility and bioavailability. To overcome these limitations, researchers have developed various formulations such as nanoformulations, solid dispersions, and microspheres. These advancements have led to improved therapeutic effects and have facilitated the progression of clinical research, primarily focusing on Phase I and Phase II trials for conditions like diabetes, obesity, and metabolic syndrome. In recent years, there has been a noticeable increase in Phase III and IV clinical trials, particularly concerning oral and dental diseases and arthritis. This article reviews recent literature from both domestic and international sources, providing a comprehensive overview of curcumin’s research progress, including its pharmacological mechanisms, formulation developments, and clinical studies.

## 1 Introduction

Curcumin is a type of polyphenolic compound extracted from the rhizomes of plants in the ginger family. Due to its bright orange-yellow appearance and superior antioxidant capabilities compared to alpha-tocopherol, curcumin is commonly utilized as a dye and culinary spice in many countries, especially in South Asia and India (Hu et al., 2017). Extensive basic research has demonstrated that curcumin possesses various pharmacological effects, including anticancer, anti-inflammatory, and antioxidant properties. However, the low water solubility, poor absorption, and rapid metabolism of curcumin present a challenge to achieving therapeutic blood concentrations after ingestion, thereby limiting its clinical application (Naksuriya et al., 2014). Scholars are currently improving the therapeutic efficacy of curcumin by developing formulations such as nanoformulations, solid dispersions, and liposomes. With the enhancement of its pharmacological effects, there has been an increase in clinical research both domestically and internationally. This article summarizes the pharmacological mechanisms, formulation research progress, and domestic and international clinical research progress of curcumin.

**FIGURE 1:**
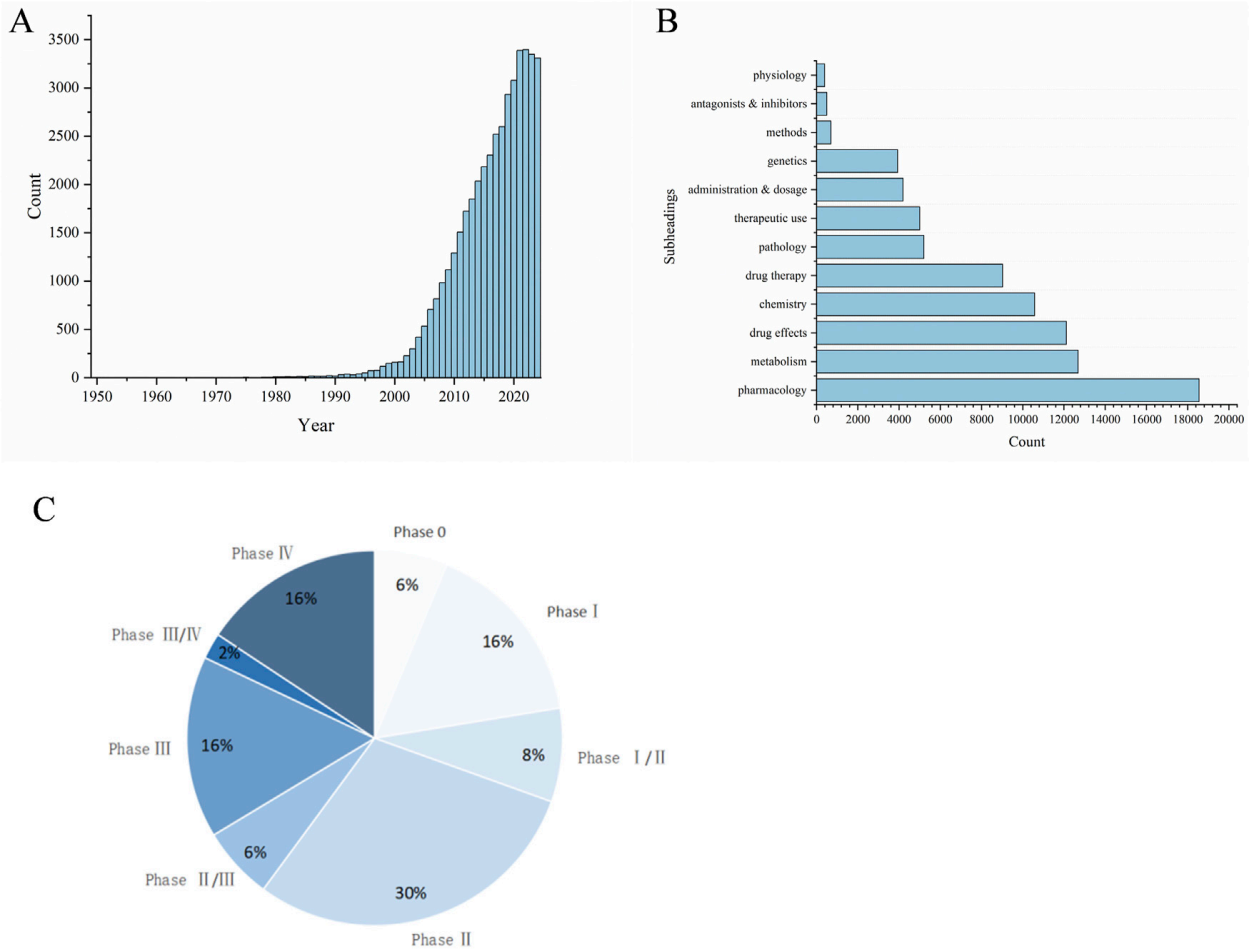
Bibliometric analysis about curcumin. **(A)** Global trends in publications about Curcumin, **(B)** Subheadings in publications about Curcumin, **(C)** Curcumin clinical period distribution.

A literature search was conducted on databases including Science Direct, PubMed, Web of Science, Springer, wiley, ACS, RSC, Google Scholar and CNKI, covering publications from the inception of these platforms up to the year 2024. Utilizing “curcumin” as the search term revealed a total of 40,775 articles, including both Chinese and English literature. A total of 25,318 articles were published in English and 15,437 in Chinese. As depicted in Figure 1A, the interest in curcumin research has surged notably in recent years. Research often focuses on pharmacology, metabolism, drug therapy, etc (Figure 1B). The focus of researches are on the antioxidant (13.8%), anticancer (10.4%), and anti-inflammatory (9.3%) attributes of curcumin, alongside the exploration of innovative formulations, including nanoparticles (8%). This review search utilized the following keywords: Curcumin, Pharmacological Effect, Formulation and Clinical research. We have summarized the above content, prioritizing authoritative and valuable literature, and this review compiles a total of 184 relevant documents.

## 2 Pharmacological actions and mechanisms of curcumin

### 2.1 Anti-cancer properties

**FIGURE 2:**
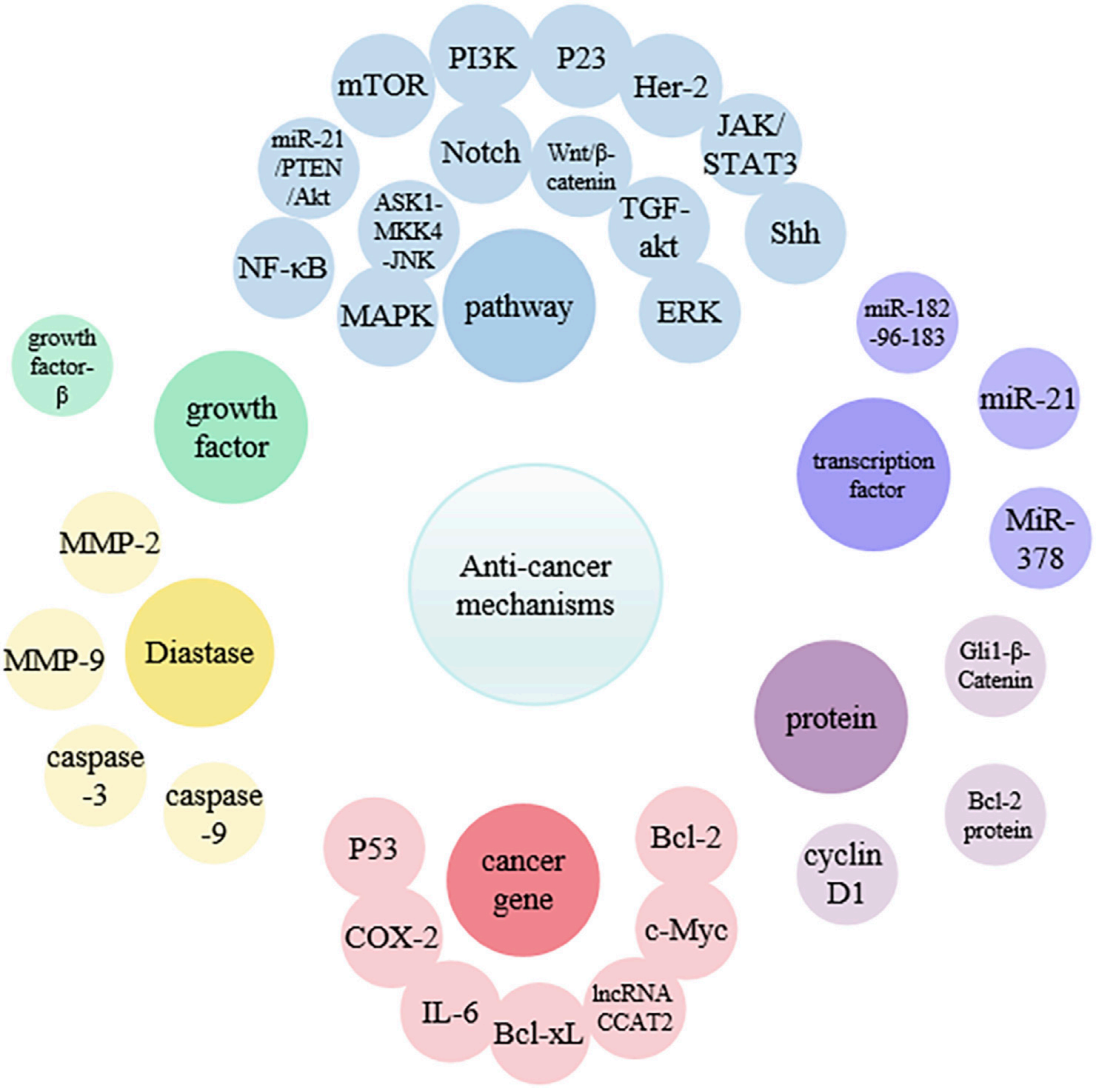
The mechanism of anti-cancer action of curcumin.

Curcumin exhibits strong therapeutic potential against various cancers in the blood, oral cavity, skin, lungs, pancreas, and intestines (Adahoun et al., 2017). In normal cells, there is a precise balance between signals that promote growth and those that inhibit growth, leading to orderly proliferation and differentiation only when necessary. However, in cancer cells, this process is completely disrupted, causing uncontrolled cell proliferation. In addition, cancer cells may exhibit metastasis, angiogenesis, and apoptosis inhibition (Hahn and Weinberg, 2002; Sa and Das, 2008). The research indicated that curcumin can effectively inhibit metastasis at various stages of the cascade, including invasion of the primary site, circulation in the blood or lymphatic vessels, extravasation at specific sites, and growth into new lesions (Wu et al., 2021) (Figure 2) (Table 1). Curcumin can target multiple molecular compounds to exert anti-metastatic effects, aciting by inhibiting NF-κB/VEGF signaling pathways to suppress angiogenesis (Huang et al., 2017). Moreover, curcumin regulates growth factors, transcription factors, kinases, enzymes, as well as pro-apoptotic and anti-apoptotic proteins, targeting cancer cell signaling pathways (such as Rb, p23, MAPK, PI3K/Akt, JAK/STAT, Shh and NF-κB), thereby promoting cancer cell apoptosis to achieve anti-tumor effects (Yu et al., 2011; Liang et al., 2014; Liang et al., 2015; Wang et al., 2018; Li et al., 2019; Qiang et al., 2019; Zhang et al., 2020; Shetty et al., 2021; Ham et al., 2022; Jabbari et al., 2022; Mishra et al., 2023). Additionally, curcumin can induce cell cycle arrest by downregulating the mRNA and protein expression of cell cycle protein D1, inhibiting the transition of cells from G (1) to S phase (Cai et al., 2009). This mechanism leads to cell cycle arrest, ultimately suppressing cancer cell growth.

**TABLE 1:** Pharmacological actions and mechanisms of curcumin.

Pharmacological effects	Curcumin dosage	Cell Type/Model type	Target molecule (+/−)	References
Anti-cancer	30 μM	HUVEC, HGC-27	NF-κB/VEGF ↓	Huang et al. (2017)
	20 μM	MKN1, MKN74, SNU668	JAK/STAT3 ↓	Ham et al. (2022)
	1–40 μmol/L	SGC-7901	NF-κB ↓	Yu and Reports (2011)
	30 μM	SGC-7901	Shh ↓, Wnt ↑, Gli1 and β-catenin Interaction ↓	Zhang et al. (2020)
	40.88 μM	AGS	c-Myc ↓	Jabbari et al. (2022)
	5–40 μM	MGC-803	miR-21/PTEN/Akt Pathway ↓	Qiang et al. (2019)
	0-20uM	BGC-823	ROS-mediated ASK1-MKK4-JNK Stress Signaling	Tao et al. (2014)
	50-100uM	SGC-7901 BGC-823 MKN1 MGC-803	PAK1 ↓ CyclinD1 ↓	Cai et al. (2009)
	5, 10, 20 μM	A549	Wnt/β-catenin ↓	Wang et al. (2018)
	50或100 mg/kg,12 weeks	BALB/c mice expose to Tobacco smoke	TS-induced MAPK/AP-1 activation ↓	Liang et al. (2015)
Anti-inflammatory	10 μM (RAW264.7) 40 μM (Caco-2)	RAW264.7, Caco-2	NO, IL-6, TNF-a, MDA, IgE, ICAM-1 ↓ IL-10, SOD ↑	Wang et al. (2023)
	50 mg/kg/d, 14 days	UUO mice	BUN, Scr ↑ IL-6, IL-1β, TNF-α ↓	Wang et al. (2020)
	10 ng/mL	HK-2 cells		
Antioxidant	0, 5, 10, 20 μM	RAW264.7 cells	SOD, CAT, GSH-PX, Nrf2 ↑	Lin et al. (2019)
	5–40 μM	LO2 cells	miR-125b-5p ↓ SOD, CAT ↑	Lin et al. (2022)
	5, 10, 20 μM	PC12 cells	SOD, CAT, GSH-PX, T-AOC ↑	Chen (2019)
	50, 100, 200 mg/kg/d, 30 d	SPF mice		
	—	LO2 cells	let-7d-5p ↑, miR-22-3p ↓	Li (2020)
Antiviral	40, 80 μM	HEK-293T	Tat degradation ↓	Ali and Banerjea (2016)
	40 μM	—	HIV-1 integrase ↓	Mazumder et al. (1995)
	10–40 μM	HeLa	dysregulation of the ubiquitin-proteasome system	Si et al. (2007)
	1, 5, 10 μM	MAGI	Tat-induced long terminal repeat region transactivation	Zhang et al. (2011a)
	5, 10, 20, 30 μM	A549, MDCK, HeLa	disrupt F-actin, viral inclusion body formation ↓	Zhang et al. (2021)
	5–15 mM	Huh7, Rep-Feo	Akt-SREBP-1 ↓	Kim et al. (2010)
Anti-Fibrotic	100, 200, 400 mg/kg	Male SD rats	PPARα ↑, TTC3/SMURF2/SMADs, EMT ↓	Kong et al. (2020)
	200 mg/kg	liver-specific autophagy deficient mice		
	10, 20, 30 μM	BNL CL.2 cells		
	200 mg/kg/d, 4 w	isoproterenol-induced cardiac hypertrophy and fibrosis in the rat	mTOR/autophagy ↓	Liu et al. (2018)
	50 mg/kg/d, 14d	male C57BL/6 mice after undergoing unilateral ureteral obstruction operations	TLR4/NF-κB, PI3K/AKT ↓	Wang et al. (2020)
Hypoglycemic	0.1, 1, 5, 10, 20, 40 µM	Huh-7/SRE-Luc cells	SREBP expression ↓	Ding et al. (2016)
	40, 80 mg/kg/d, 12 w	Male C57BL/6 mice		
	0.2 g/kg/d, 6 w	C57BL/KsJ db/db mice, lean heterozygote non-diabetic db/+	the hepatic activities of fatty acid synthase, beta-oxidation, 3-hydroxy-3-methylglutaryl coenzyme reductase, and acyl-CoA: cholesterol acyltransferase ↓	Seo et al. (2008)
	1, 5, 20, and 50 μg/mL	Caco-2 cells	α-glucosidase ↓	Liu et al. (2024a)
	250 mg/kg/d, 8 w	T2DM rat models	the blood glucose, AUC of glucose level, FFA, TNF-α ↓	Su et al. (2017)
Enzyme inhibition activity	10, 20 μM	NIH-3T3 cells	the enzyme steroid sulfatase ↓	Balasubramonian and Selcer (2023)
	30 μM	*Escherichia coli*	F1 ATPase ↓	Sekiya et al. (2014)
	2–20 μM	RPE.40, CHO, HT-29, SW480 and Caco-2 cells	pro-IGF-1R, pro-GPC3, pro-GPC4 ↓	Zhu et al. (2013)
	0.04–5 μM	—	GSTs A1-1, A2-2, M1-1, M2-2 和 P1-1 ↓	Hayeshi et al. (2007)

### 2.2 Anti-inflammatory properties

**FIGURE 3:**
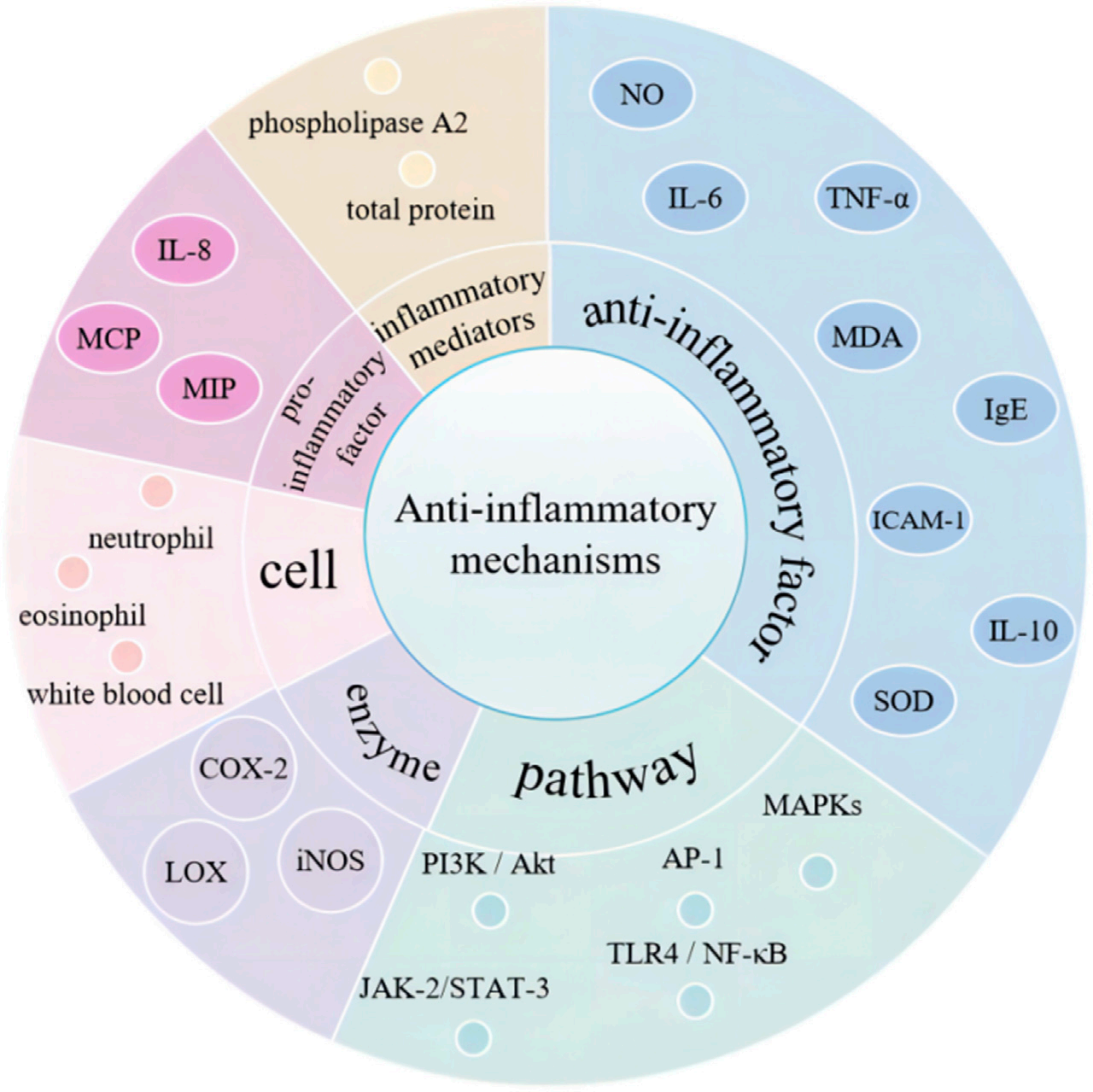
The anti-inflammatory mechanism of curcumin.

The anti-inflammatory properties of curcumin are primarily attributable to it capacity to inhibit pro-inflammatory factors. The inflammatory responses are regulated by blocking the expression of pro-inflammatory cytokines such as IL-8, MCP, and MIP through the modulation of signaling pathways, enzymes, and inflammatory mediators (Pontes-Quero et al., 2021) (Figure 3). Wang et al. have demonstrated that curcumin can suppress the expression of inflammatory factors, including NO, IL-6, TNF-a, MDA, IgE, and ICAM-1. Simultaneously, curcumin enhances the levels of IL-10 and SOD, thereby exerting its anti-inflammatory properties and offering a potential therapeutic approach for airway inflammation (Wang et al., 2023). Curcumin has been shown to reduce the production of pro-inflammatory factors, including IL-1, IL-6, IL-8, and TNF-α, by modulating the TLR4/NF-κB/AP-1 signaling pathway. This action contributes to the mitigation of intestinal inflammation in patients suffering from inflammatory bowel disease (Gong et al., 2018). Furthermore, curcumin also inhibits epithelial-mesenchymal transition and inflammatory responses through the suppression of the TLR4/NF-κB and JAK-2/STAT-3 signaling pathways (Wang et al., 2020). Cyclooxygenase-2 (COX-2), lipoxygenase (LOX) and inducible nitric oxide synthase (iNOS) are instrumental in the mediation of the inflammatory process. The inhibitory effects of curcumin on these enzymes play a pivotal role in regulating the inflammatory response (Menon and Sudheer, 2007). Additionally, curcumin has been demonstrated to reduce the number of white blood cells, neutrophils, and eosinophils, exerting a protective effect on the levels of serum inflammatory mediators in various inflammatory diseases, such as phospholipase A2 and total protein (Memarzia et al., 2021).

### 2.3 Antioxidant properties

**FIGURE 4:**
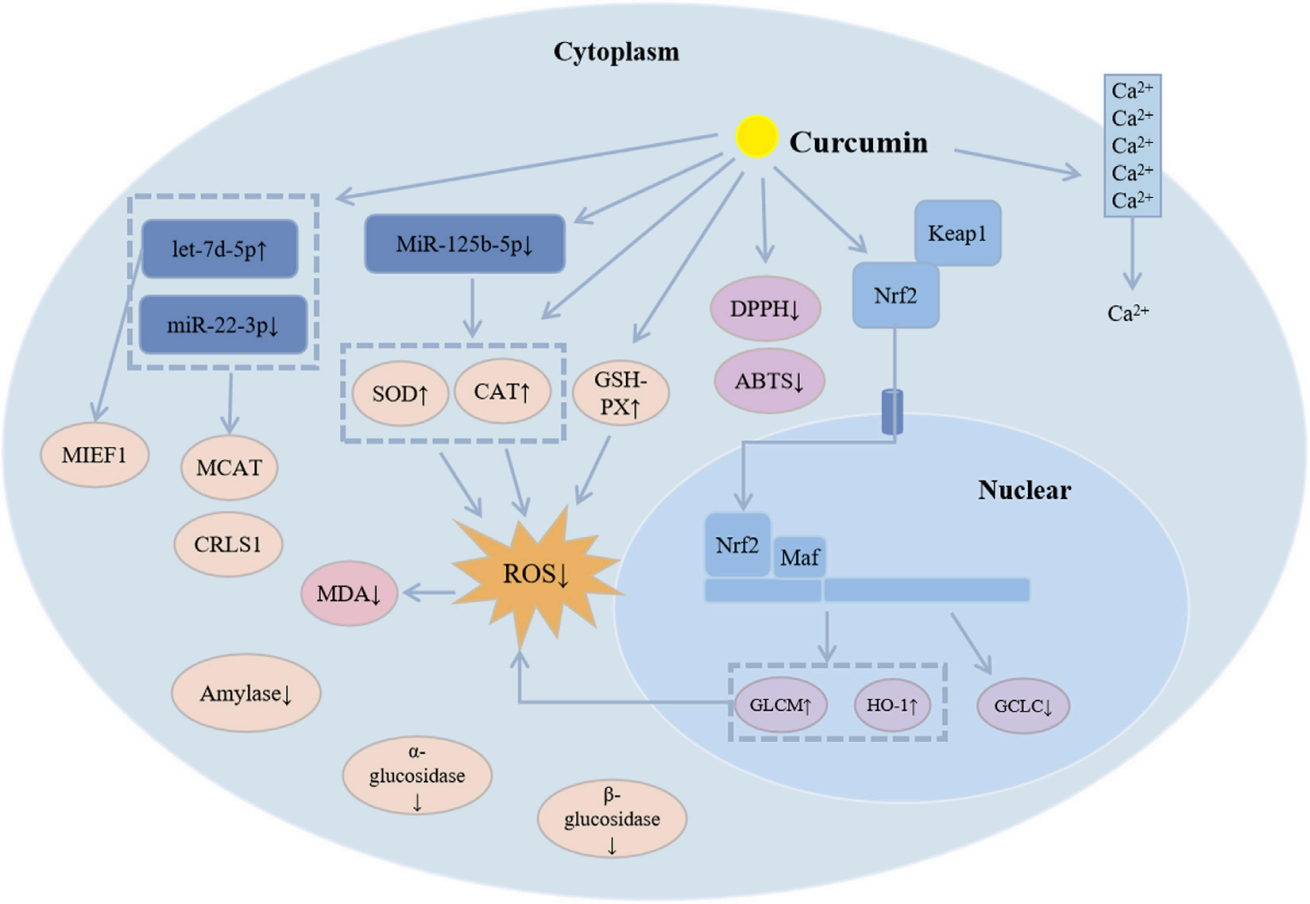
The antioxidant mechanism of curcumin.

Curcumin proficiently eliminates excess reactive oxygen species (ROS) in the body while simultaneously enhancing the activity and expression of antioxidant enzymes, thereby augmenting overall antioxidant capacity (Figure 4). Studies have shown that curcumin can enhance cellular ROS elimination by increasing the activities of catalase, superoxide dismutase, and glutathione peroxidase while decreasing the activities of amylase, α-glucosidase, and β-glucosidase (Balasubramanyam et al., 2003; Lin et al., 2019; Kanwal et al., 2023). It also interferes with protein kinase C activity and inhibits Ca^2+^ influx. Additionally, curcumin exhibits antioxidant properties by scavenging DPPH and ABTS free radicals and reducing lipid peroxidation through increased catalase activity, thereby alleviating oxidative stress (Chen et al., 2018). The Keap1-Nrf2 pathway is a key antioxidant system, and curcumin is capable of regulating this pathway to maintain the cell’s redox balance and eliminate ROS (Ghareghomi et al., 2021). Furthermore, curcumin may significantly enhance the cell’s antioxidant capacity by suppressing the expression of miR-125b-5p (Lin et al., 2022).

Curcumin enhances the expression of mitochondrial microRNAs, notably let-7d-5p, while concurrently reducing the levels of miR-22-3p. Subsequent investigations indicate that let-7d-5p specifically targets mitochondrial elongation factor 1 (MIEF1), whereas cardiolipin synthase 1 (CRLS1) and malonyl-CoA-acyl carrier protein transacylase (MCAT) are targeted by miR-22-3p. Both let-7d-5p and its target MIEF1, along with miR-22-3p and its targets CRLS1 and MCAT, are instrumental in modulating cellular antioxidant capacity and mitochondrial functionality, with their interactions producing contrasting effects (Li, 2020). Additionally, curcumin may contribute to the maintenance of mitochondrial homeostasis by regulating mitochondrial biogenesis, which supports the stability of the body’s redox system and ultimately mitigates oxidative stress (Chen, 2019).

#### 2.3.1 Gastrointestinal diseases

Oxidative stress in inflammatory bowel disease (IBD) results from an imbalance between oxidants and antioxidants within the affected tissues (Sahoo et al., 2023). Curcumin promotes Parkin-dependent mitophagy by inducing AMPK (AMP-activated protein kinase) and facilitating the nuclear translocation of TFEB (transcription factor EB). This process not only alleviates oxidative stress but also strengthens the integrity of the intestinal barrier and mitochondrial function. Furthermore, curcumin reduces the generation of ROS and mitigates LPS-induced inflammation in vascular smooth muscle cells through the TLR4-MAPK/NF-κB signaling pathway.

#### 2.3.2 Ocular diseases

The accumulation of ROS is commonly linked to a range of retinal diseases, such as cataracts, age-related macular degeneration, uveitis, diabetic retinopathy, retinal tumors, and proliferative vitreoretinopathy (PVR) (Maiuolo et al., 2022). To explore the protective effects of curcumin against oxidative stress-related damage, Raman et al. found that curcumin prevents apoptosis in human retinal epithelial cells. This protection is achieved through several mechanisms, including the inhibition of lipid peroxidation in lens membranes, the induction of antioxidant enzymes, the regulation of calcium homeostasis in the lens, the support of lens chaperones, and the modulation of transcription factor activity within the lens (Raman et al., 2016).

#### 2.3.3 Liver diseases

Curcumin exhibits both preventive and therapeutic effects against oxidative-related liver diseases, such as hepatotoxicity, non-alcoholic fatty liver disease, alcoholic liver disease, liver fibrosis, cirrhosis, and liver injury. These effects are mediated through various cellular signaling pathways. Notably, these include the ERK/p38/MAPK pathway, the Nrf2/ARE/Keap1 signaling cascade in the liver, and the upregulation of detoxification gene expression. Furthermore, Curcumin influences TIMP signaling, the AMPK pathway, lipid metabolism, and the downregulation of Rac1, NOX1, and Rac1-GTP signaling (Farzaei et al., 2018).

#### 2.3.4 Neurodegenerative diseases

The emergence and progression of neurodegenerative diseases (NDD) such as Alzheimer’s disease (AD), Parkinson’s disease (PD), and Huntington’s disease (HD) are intricately associated with oxidative stress. Curcumin plays a significant role in mitigating this stress by inhibiting the oxidation of lipids and proteins, which effectively reduces levels of malondialdehyde, protein carbonyls, thiols, and nitrotyrosine. Furthermore, curcumin enhances the activity of crucial antioxidant enzymes, including superoxide dismutase and glutathione peroxidase, thereby potentially decelerating or ameliorating the trajectory of NDD (Abrahams et al., 2019).

#### 2.3.5 Cardiovascular diseases

Curcumin plays a pivotal role in combating cardiovascular diseases by modulating superoxide dismutase, heme oxygenase-1, and nuclear factor erythroid 2-related factor 2. This modulation improves arterial health, especially by enhancing endothelial function and reducing arterial stiffness, thereby lowering the risk of cardiovascular diseases (CVD) (Fleenor et al., 2019).

### 2.4 Antiviral properties

**FIGURE 5:**
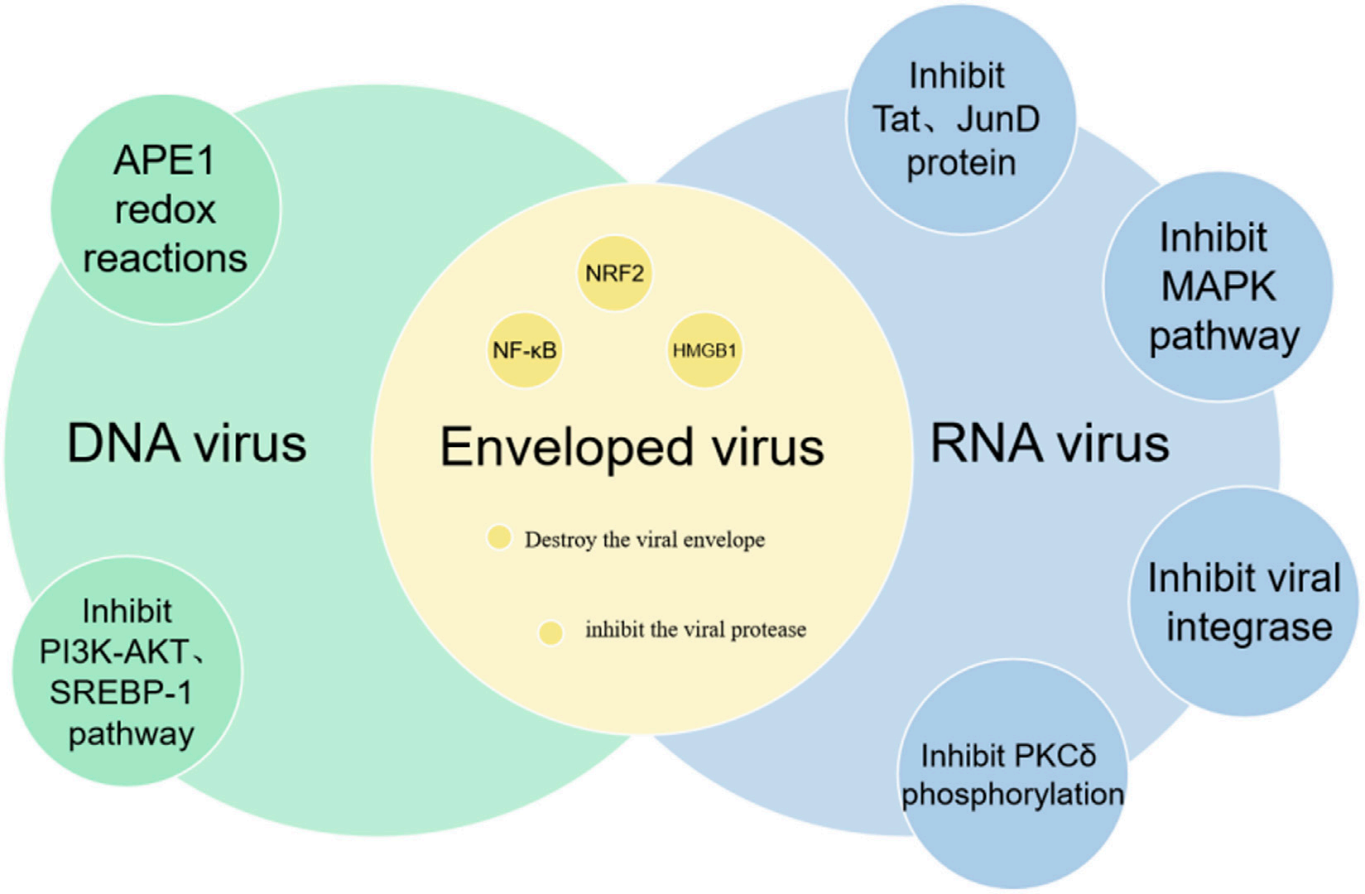
The antiviral mechanism of Curcumin.

Curcumin, the active compound in turmeric, has been demonstrated to possess antiviral properties against both DNA and RNA viruses (Figure 5). Research has indicated that curcumin can inhibit DNA viruses by suppressing the APE1 redox reaction, and it can target RNA viruses by inhibiting viral integrase, proteases, and reducing the activity of Tat and JunD proteins (Mazumder et al., 1995; Si et al., 2007; Zhang H. S. et al., 2011; Ali and Banerjea, 2016). Zhang et al. found that curcumin can disrupt the F-actin in HPIV3, inhibiting the formation of viral inclusion bodies (IB) and suppressing viral replication (Zhang et al., 2021). Additionally, curcumin downregulates PI4KB, preventing its co-localization within the viral IB. Kim et al. demonstrated that curcumin can inhibit the replication of hepatitis C virus by suppressing the PI3K/Akt and SREBP-1 pathways (Kim et al., 2010). Further studies (Thimmulappa et al., 2021) have revealed that curcumin can directly interact with the envelope proteins of enveloped viruses, disrupt the viral envelope, inhibit viral proteases, and modulate host factors such as NF-κB, NRF2 and HMGB1, all of which contribute to its broad-spectrum antiviral activity.

### 2.5 Anti-fibrotic properties

The anti-fibrotic effects of curcumin have been demonstrated in a number of organs, including the liver, heart and kidneys. The underlying mechanisms may differ depending on the type of fibrosis. Kong et al. found that curcumin can inhibit hepatic fibrosis by suppressing epithelial-mesenchymal transition (EMT) in hepatocytes and by activating autophagy to inhibit the production of extracellular matrix (Kong et al., 2020). Liu et al. have demonstrated that curcumin can alleviate isoproterenol-induced cardiac hypertrophy and fibrosis by inhibiting autophagy and activating the mTOR pathway (Liu et al., 2018). Wang et al. have also shown that curcumin can attenuate obstructive nephropathy-induced renal interstitial fibrosis by suppressing EMT through the inhibition of the TLR4/NF-κB and PI3K/AKT signaling pathways (Wang et al., 2020).

### 2.6 Hypoglycemic properties

The primary mechanism by which curcumin exerts its hypoglycemic effects is through the attenuation of insulin resistance. Studies have demonstrated that curcumin can combat insulin resistance by increasing the levels of circulating adiponectin and leptin, activating PPARγ, inhibiting Notch1 signaling, and regulating SREBP target genes (Ding et al., 2016; Balakumar et al., 2023). Furthermore, studies have demonstrated that curcumin can significantly reduce blood glucose and glycated hemoglobin levels, improve glucose homeostasis, and enhance plasma insulin levels (Seo et al., 2008). Research has demonstrated that curcumin reduces α-glucosidase (α-Glu) activity (Liu L. et al., 2024) and inhibits aminopeptidase B activity (Cadel et al., 2019), which is expected to become a potential therapeutic method for the treatment of diabetes. Additionally, experiments have shown that curcumin can ameliorate glucose and lipid metabolism disorders, increase insulin sensitivity, and improve insulin resistance in animal models of type 2 diabetes mellitus (T2DM) (Su et al., 2017).

### 2.7 Enzyme inhibition activity

Curcumin exhibits significant potential in the treatment of various diseases through the inhibition of multiple enzymes. Research demonstrates that curcumin effectively suppresses steroid sulfatase activity, resulting in decreased levels of active steroid hormones, such as estrogen and androgen, within tissues. This mechanism positions curcumin as a promising therapeutic agent for estrogen-dependent cancers and other related conditions (Balasubramonian and Selcer, 2023). In addition, curcumin inhibits F1-ATPase activity by interfering with the conformational changes of the β subunit’s catalytic site, thereby extending the duration of catalytic activity (Sekiya et al., 2014). It also diminishes the activity of glutathione S-transferase (GST), which may reduce resistance to chemotherapy agents (Hayeshi et al., 2007). Furthermore, curcumin impedes the maturation of proprotein convertase (PC) zymogens, leading to a suppression of PC activity and influencing calcium (Ca^2+^) uptake into the endoplasmic reticulum (Zhu et al., 2013). Moreover, studies indicate that curcumin lowers the apparent affinity of Na,K-ATPase for potassium (K^+^) while enhancing its affinity for sodium (Na^+^) and ATP (Mahmmoud, 2005). Additionally, aminopeptidase B (Ap-B), a Zn^2+^-aminopeptidase from the M1 family, plays a vital role in the maintenance of glucose homeostasis.

## 3 Curcumin *in silico* studies

To facilitate further exploration of the therapeutic potential of curcumin, one can conduct target prediction through the analysis of its physicochemical parameters and structural similarities, thereby evaluating other possible molecular targets. Rahman et al., who conducted docking studies to examine the interactions of curcumin with GSTA1, GSTO1, KEAP1, BACE1, and MAOA (Rahman et al., 2022). Concurrently, Singh et al. investigated curcumin’s interactions with COX-2, 5-LOX, TNF-α, and IL-6 (Singh et al., 2023) to identify potential molecular targets for its antioxidant properties. Computational analyses indicate that curcumin is likely to engage with GSTA1, GSTO1, and KEAP1, thereby enhancing its antioxidant efficacy. In contrast, its binding to BACE1 and MAOA may contribute to the mitigation of oxidative damage (Rahman et al., 2022). Notably, curcumin demonstrates a strong affinity for the enzyme COX-2 (Singh et al., 2023). Throughout the simulation process, curcumin exhibited excellent binding stability and minimal fluctuations when interacting with additional targets, including 5-LOX, TNF-α, and IL-6.

Recent research has docked curcumin with critical compounds of the Wnt/β-catenin signaling pathway, particularly casein kinase-1 (CK1) and glycogen synthase kinase-3 (GSK3B). Findings suggest that curcumin may exert its antimicrobial effects via this pathway (Chopra et al., 2023). Moreover, curcumin has demonstrated effectiveness against *Staphylococcus aureus*, a common pathogen. Studies indicate that the formation of its biofilm is significantly influenced by the polysaccharide intercellular adhesion (PIA), which is regulated by the icaADBC operon (Khaleghian et al., 2023). This bacterium can express various fibronectin-binding proteins (FnbA) and clumping factor A (ClfA), which facilitate bacterial adhesion to surfaces and contribute to biofilm development. The accessory gene regulator (Agr) system also plays a vital role in regulating numerous pathogenic factors, including biofilm formation. This system, consisting of AgrB, AgrC, AgrA, and AgrD, is crucial for modulating the virulence factors of *Staphylococcus aureus*. Notably, curcumin exhibits a strong binding affinity for these genes, indicating its potential as a novel therapeutic agent against *Staphylococcus aureus*.

Furthermore, Patil et al., who investigated the interactions of curcumin with anti-inflammatory targets such as IL-6, TNF-α, NF-κB, and SIRT1. Their findings revealed significant binding interactions with IL-6, TNF-α, and SIRT1, while the interaction with NF-κB was comparatively weaker (Patil et al., 2024). Similarly, Selim et al. performed docking studies with P38α, MAPK, ERK1/2, JNK1, and MK3, which showed favorable binding energies, suggesting these could be potential anti-inflammatory targets for curcumin (Selim et al., 2019). In addition, other researchers have examined curcumin’s interactions with gene targets related to metabolic syndrome, diabetes, and cognitive disorders, reporting promising binding results (Nguyen and Kim, 2022; Vaithiyalingam et al., 2023) (Table 2).

**TABLE 2:** Curcumin *in silico* studies.

Pharmacological effects	Gene	Amino acid binding sites	Binding energy (Kcal/mol)	References
Anti-oxidation, anti-aging	COX-2	SER 530, SER 353, SER 119, LYS 83, ARG 120, VAL 89, LEU 93, TRP 100, ILE 112, TYR 115, VAL 116, PHE 205, VAL 344, TYR 348, VAL 349, LEU 352, TYR 355, PHE 381, LEU 384, TYR 385, TRP 387, MET 522, VAL 523, ALA 527, LEU 531, LEU 534	−8.500	Singh et al. (2023)
	5-LOX	ASP 170, ARG 401, ALA 606, GLU 612, GLN 15, ASN 180, GLN 609, GLN 611, ASN 613, LYS 183, ARG 401, GLU 614, VAL 175, ALA184, PHE 402, TRP 605, ALA 606, LEU 615, ILE 673	−3.963	
	TNF-a	GLN 61, TYR 59, SER 60, LEU 57, IL3 58, TYR 151, ILE 155	−5.283	
	IL-6	THR 556, SER 558, HIS 552, THR 553, ASP 549, GLU 554, VAL 514, VAL 548, TYR 555, LEU 557	−3.610	
	BACE1	GLY72, GLN73, ILE171, TYR132, Ser71, GLY74, ARG296	−8.5	Rahman et al. (2022)
	GSTA1	GLY14, THR68, ILE106, LEU107, MET208, ALA100	−9.6	
	GSTO1	CYS32, PRO33, PHE34, LEU56, VAL72, PRO124, ILE131, TRP180, TYR229	−8.6	
	KEAP1	GLY603, GLY364, GLY509, ILE416, LEU365, ALA510, VAL512, VAL463, VAL418, VAL465, THR560, ILE559, VAL606, GLY558, LEU557, GLY605, GLY464, GLY462, VAL604, SER508, ALA556, TYR525, ILE559, VAL606, ARG415	−8.6	
	MAOA	GLY55, GLY56, ALA57, TYR58, MET339, GLN204, PHE97, LEU326, SER198, TYR433, ILE169, ILE324, PHE197, ILE314, GLY99, TYR396, CYS312, LEU86, ALA100, VAL199, LEU86	−9.2	
antibacterial	CK1	PRO87, LEU85, ILE148	−6.9875	Chopra et al. (2023)
	GSK3B	VAL135	−6.5164	
Anti-staphylococcus aureus	*Staphylococcus aureus*	SER412, SER351, PHE306, GLN223	−52.44	Gao et al. (2023)
	IcaB	HIS51, LEU252, TYR75, TYR230, LEU6, GLY231, LYS190, SER189	−7.7	Khaleghian et al. (2023)
	IcaC	LEU14, LEU11, ARG282, LYS36, PHE286, LYS289	−6.9	
	ArgB	ASN45, PRO42, LYS7, LYS212, LEU175, GLY10, ALA157, ARG62, SER142, LEU61, ILE155	−7.0	
	ArgC	SER36, SER34, HIS35, SER10, TYR12, AGR183, CYS147, ALA72, ALA316, LEU95	−7.6	
	FnbA	ASN304, PHE306, VAL256, HIS220	−7.2	
	CfbA	PRO251, ASP385, VAL288, THR397, PRO341, TYR436, PHE455, ARG395, ASN284	−7.8	
	IcaA	ILE215, GLY258, VAL371, ALA255	−8.1	
	IcaD	VAL38, ALA79, CYS35, MET75, PHE31, SER28	−8.3	
Hypoglycemic	SGLT-2	ILE253	−6.62	Vaithiyalingam et al. (2023)
	PPAR	ASN568, ALA552	−6.35	
	Alpha-glucosidase	LYS 809	−3.59	
	DPP-4	TRP216, LEU410, PHE364	−6.9	
	alpha-amylase	LYS457	−5.14	
Anti-cognitive impairment	NFKB1	ARG140, LEU346, THR179, LYS177, HIS173	−8.1	Nguyen et al. (2022)
	IL1B	VAL41, LYS27, LEU62, VAL19, LYS63, VAL40	−6.8	
	IL6	ARG182, ARG179	−5.7	
	IL10	PHE30, LEU26, LEU98, TYR72	−6.6	
	TNF	GLU104, GLN102, GLU116, LYS98	−7.8	
Metabolic syndrome	PPARG	LYS457, LEU465, LYS474, ASP475, GLN454	−5.8	Nguyen and Kim (2022)
	INS	SER809, LEU217, GLU813, LEU817, SER409, GLU213, HIS210, GLU413, GLU613, ALA214	−8.3	
	IL6	ARG182, ARG179	−5.6	
	CRP	GLU130, PRO29, THR126, ARG58	−4.9	
Anti-inflammatory	P38 α MAPK	Gly110, Gly033, Met109, Thr106, Asp168, Asn155, His10	−24.639	Selim et al. (2019)
	ERK1/2	MET108, Gln105, Glu33, Thr110, Lys114	−21.8	
	JNK1	ASN114, Met111, Lys55, Ile32	−17.72	
	MK3	Lys73, Met121, Glu119, Glu125	−24.63	
	IL6	Thr130, Thr134	−5.7	Patil et al. (2024)
	TNF-α	Asn134, Asn148	−5.5	
	NF-κB	DC614, DC615	−4.7	
	SIRT1	Pro211, Glu214, Asp298	−7.1	

It is essential to recognize that not all docking results are positive. For example, curcumin does not effectively bind to the ATP binding sites of CDK2, CDK4, and CDK6. This suggests that curcumin is unlikely to inhibit the CDK pathway in cancer, indicating that it may influence cancer growth through alternative mechanisms (Ahmed et al., 2017).

## 4 Research on curcumin formulations

### 4.1 Nanoparticles

In recent years, there has been a notable increase in the development of biodegradable nanoparticles. These nanoparticles are produced using a variety of materials, including polysaccharides such as chitosan, alginate, and starch (Maghsoudi et al., 2017), as well as proteins such as walnut protein, zein, and sodium caseinate (Li et al., 2022; Liu et al., 2022; Lv et al., 2023), and polymers such as sulfated κ-carrageenan (Nogueira et al., 2022). The curcumin-nanoparticles described above have been demonstrated to improve the solubility, bioavailability, and biological properties of curcumin. It has been demonstrated in studies that polysaccharide-based curcumin nanoparticles, such as those made with chitosan, starch, and alginate, have minimum inhibitory concentrations against *Streptococcus* mutans that are more than twice as effective as free curcumin (Maghsoudi et al., 2017). Phospholipid and zein-coated curcumin nanoparticles exhibit excellent aqueous dispersibility across a broad pH range (Liu et al., 2022). Additionally, this encapsulation substantially improves the stability of curcumin against heat and enhances its antioxidant properties. *In vitro* release experiments reveal that these lipid-coated nanoparticles effectively inhibit the leakage of curcumin in the stomach, thereby enhancing its oral delivery efficiency to the small intestine.

In order to further enhance the therapeutic efficacy of curcumin, researchers have explored the possibility of developing targeted nanoparticles. Fan et al. designed a novel PLGA-PEG-B6 peptide-conjugated nanoparticle loaded with curcumin (PLGA-PEG-B6/CUR) and demonstrated its efficacy in enhancing spatial learning and memory in APP/PS1 transgenic mice, as well as reducing the formation and deposition of amyloid-β and tau hyperphosphorylation in the hippocampus (Fan et al., 2018). Hong et al. employed microfluidic-assisted continuous precipitation to prepare enzyme-targeted curcumin-loaded nanoparticles (CUR-P-NPs), which exhibited enhanced cellular uptake, antiproliferative effects on U251 cells, and tumor-targeting abilities compared to curcumin solution and non-targeted curcumin nanoparticles (Hong et al., 2021). However, these nano-targeted materials lack standardized production and quality control measures, which complicates the testing of their efficacy and safety. As a result, these formulations remain in the laboratory stage and face significant hurdles in advancing to clinical trials.

### 4.2 Nanoemulsions

Nanoemulsions typically consist of an aqueous phase, an oil phase, and surfactants, with the potential inclusion of co-surfactants. Research have demonstrated that nanoemulsification can significantly improve the permeability of curcumin, resulting in a shift from a zero-order release profile to a Higuchi release profile (Rachmawati et al., 2015). Yousef et al. developed a nanoemulsion with biocompatible compounds (surfactant glycerides, lecithin, and co-surfactants ethyl ether and ethanol) to enhance the skin delivery of curcumin (Yousef et al., 2019). This approach offers a novel opportunity for the targeted treatment of psoriasis and skin cancer. It is crucial to evaluate how the parameters of nano-system formulations affect skin permeability. Additionally, it is recommended to address any complications that may arise from non-Newtonian flow behavior. Kumari et al. developed a nanoemulsion that enhanced the dermal penetration and antimicrobial activity of curcumin, thereby suggesting its potential as a topical antimicrobial agent for the treatment of burn wound infections (Kumari and Nanda, 2023). The droplet size of nanoemulsions, which typically ranges from 20 to 500 nm, can influence the bioaccumulation of curcumin. Shen et al. prepared nanoemulsions with varying mean diameters (150 nm and 300 nm), lipid compositions (MCT, corn, and fish oils), and emulsifiers (Tween 80 and whey protein), and observed that the bioaccumulation of curcumin increased with particle size, exhibiting higher levels in corn oil-based nanoemulsions relative to fish oil or MCT-based ones (Shen et al., 2019). Additionally, greater accumulation was noted in whey protein-stabilized nanoemulsions compared to Tween 80-stabilized ones. Several nanoemulsions have already been launched in the field of ophthalmology, showcasing notable benefits in improving drug stability and bioavailability. Consequently, curcumin nanoemulsions show potential for progression into clinical trials as a promising new treatment option for various conditions.

### 4.3 Nanocrystals

Curcumin nanocrystals are typically composed of the active compound and stabilizers, exhibiting low toxicity and high structural stability. Lizoňová et al. prepared curcumin nanocrystals using a combination of steric and ionic stabilizers (Tween 80, sodium dodecyl sulfate, Poloxamer 188, hydroxypropyl methylcellulose, polyethylene glycol, and phospholipids) via wet milling (Lizoňová et al., 2022). The resulting nanocrystals were found to have colloidal stability in culture media and to be non-toxic to healthy cells. The local application of curcumin nanocrystals is also feasible, as the increased kinetic solubility of nanocrystals can lead to a concentration gradient that promotes passive skin permeation (Pelikh et al., 2021) The dermal penetration and accumulation of curcumin nanocrystals depend on the carrier, permeation enhancers, and particle size. Xiang et al. prepared curcumin nanocrystals with xanthan gum (serving as the carrier) and propylene glycol (acting as the permeation enhancer) (Xiang et al., 2023). The results showed that xanthan gum reduced the follicular penetration and passive skin accumulation of curcumin nanocrystals, whereas propylene glycol enhanced their skin penetration and retention. The results demonstrated that smaller curcumin nanocrystals exhibited higher passive skin permeability compared to larger ones. Furthermore, nanocrystals have the potential to facilitate sustained topical delivery of curcumin by targeting it to hair follicles (Pelikh et al., 2021). Consequently, curcumin nanocrystals exhibit considerable benefits in treating skin disorders, showing potential for progression into clinical trials as an innovative topical therapeutic agent.

### 4.4 Nano micelles

Nano micelles are thermodynamically stable colloidal aggregates that can passively target drugs to specific sites, enhancing their solubility and bioavailability through improved permeation and retention effects. Desai et al. prepared curcumin cocrystal micelles for nose-to-brain delivery (Desai and Patravale, 2018). The prepared micelles exhibited enhanced antioxidant activity, improved bioavailability, and significant brain distribution, indicating their potential for treatment neurodegenerative diseases. In a study by Tima et al. the thin-film hydration method was employed to prepare curcumin micelles conjugated with an FLT3-specific peptide (FLT3-CUR) (Tima et al., 2019). The FLT3-CUR micelles not only improved solubility but also enhanced anti-leukemic activity. This Curcumin-targeted micelle nanocarrier may be an effective leukemia treatment, especially for FLT3-ITD overexpressing leukemic model. Sun et al. used the thin-film dispersion method to prepare CUR-loaded GAL-PEG-PLA/TPGS micelles, which exhibited sustained-release properties (Sun et al., 2021). The high-GAL-density GPP micelles were advantageous in promoting epithelial cell uptake and improving intestinal permeability. The prepared micelles could improve relative bioavailability while reducing damage to liver and intestinal tissues. However, research on nano-micelles is still in its nascent stages. Their stability necessitates further refinement, and their biocompatibility and safety also require further verification. As a result, they are currently confined to the laboratory phase of investigation.

### 4.5 Solid dispersions

Solid dispersion is a system where curcumin is dispersed in a carrier in a highly dispersed state, such as molecular, amorphous, or microcrystalline. Research has demonstrated that the use of carriers, such as polyethylene glycol (PEG), polyvinylpyrrolidone (PVP), and Eudragit^®^ EPO (EPO) can increase the solubility, dissolution rate, and bioavailability of curcumin, leading to better therapeutic effects (Huang et al., 2008; Guan, 2009; Gao et al., 2011; Huang et al., 2018; Lu et al., 2018; Zhang et al., 2018; Liang et al., 2019; Lu and Deng, 2019; Shin et al., 2019; Zhang Q. et al., 2019; Yu et al., 2020; Fan et al., 2021; Ren et al., 2022). The extent of improvement varies depending on the carrier used. The combined use of different carriers can also have different effects. For example, the dissolution rate of PVP K30 combined with HPMC is higher than using PVP K30 alone. However, the combination of PVP-based carriers with the surfactant F68, or the combination of polyvinylpyrrolidone and poloxamer, can actually decrease the solubility and bioavailability (Pu et al., 2022; Li et al., 2023). The specific details are summarized in Table 3. In summary, the selection of carrier materials for curcumin solid dispersions is remarkably extensive, and their physicochemical properties and biocompatibility have been rigorously validated. In addition, solid dispersion technology has matured, and many solid dispersion preparations have been marketed. This approach markedly improves the solubility of curcumin while ensuring exceptional stability. As a result, curcumin solid dispersions show great potential for progressing to clinical trials aimed at treating various diseases.

**TABLE 3:** Solid dispersions of curcumin with different excipients.

Cur-SD	Result	References
Cur-PEG6000-SD	In distilled water, the dissolution rate reached 93%	Ren et al. (2022)
Cur-PVP-SD	The absorption rate of curcumin solid dispersions is 6.75 times that of curcumin suspensions	Huang et al. (2008)
Cur-PVP K30-SD	(1) The solubility was increased by 2,320-fold. (2) In pH 6.8 PBS buffer, the dissolution rate reached 72% within 30 min (3) With the addition of piperine (pip), the relative bioavailability increased by 271%, and the dissolution rate in 0.5% Tween 80 hydrochloric acid solution reached 78.21%	Gao et al. (2011), Huang et al. (2018), Lu et al. (2018), Liang et al. (2019)
Cur-PVP K29/32-SD	The relative bioavailability was 192%, and the cumulative dissolution rate in 0.1% SDS artificial gastric fluid was 98%	Guan (2009)
Cur-EPO-SD	The relative bioavailability was 145%, and the cumulative dissolution rate in pH 1.2 HCl solution was around 90% after 1 h. The dissolution rate increased to 234.2 μg/mL	Fan et al. (2021)
Cur-tryptophan-SD	In a phosphate buffer solution containing 0.5% polysorbate 80 at pH 6.8, the cumulative dissolution rate was 90.37%. Under non-leaching conditions, the solubility reached 134.81 μg/mL	Lu and Deng (2019)
Cur-HPMC-SD	(1) The water solubility of curcumin was significantly improved to 238 μg/mL, and it exhibited sustained-release properties. (2) It inhibited the release of lactate dehydrogenase and reduced the apoptosis-related proteins in t-BHP-treated HepG2 cells	Shin et al. (2019), Yu et al. (2020)
Cur-arabinogalactan-SD	The crystallinity was reduced, the solubility increased by 10.5-fold, and the bioavailability was significantly improved by 8-fold	Zhang et al. (2019a)
Cur-NAGA-SD	In U87 MG glioblastoma cells, the formulation induced higher cytotoxicity compared to free curcumin. The bioavailability was increased by 19-fold compared to free curcumin	Zhang et al. (2018)
Cur-HPMC-ecithin- isomalt-SD	The bioavailability was 13-fold that of the active pharmaceutical compound, and the anti-inflammatory efficacy of curcumin was enhanced.The bioavailability was 13-fold that of the active pharmaceutical compound, and the anti-inflammatory efficacy of curcumin was enhanced	Chuah et al. (2014)
Cur-Eudragit RS/RL-SD	With good miscibility, the relative bioavailability was 223.44%, the half-life was prolonged, and the blood concentration was stable and effective	Fan et al. (2020)
Cur-TPGS-mannitol-SD	Increased in the maximum plasma concentration (C max) and area under the plasma concentration-time curve (AUC) of 86- and 65-fold	Song et al. (2016)
Cur-PVP K30-HPMC-SD	The dissolution rate increased by 2-fold, and in 0.1% SDS-HCl and 0.15% polysorbate 80-phosphate, 80% was dissolved within 30 min	Li et al. (2023)
Cur-F127-TPGs-SD	The relative bioavailability was 705%, and in pure water, the cumulative dissolution rate reached 90.31% within 60 min, with a saturated solubility of 583.17 μg/mL	Xu et al. (2023)
Cur-EPO-HPMC-SD	In 0.5% Tween 80 pH 1.2 HCl solution, the dissolution rate could exceed 90% within 5 min, and the saturated solubility was 9.7-fold that of the active pharmaceutical compound	Zhang et al. (2019b)
Cur-PVP/VA-Poloxamer-SD	The solubility was improved to 0.230 g/L, and in 900 mL of 0.5% SDS solution, the dissolution rate was 100% within 30 min. Only the solubility of poloxamer was improved to 0.971 g/L	Pu et al. (2022)

### 4.6 Liposomes

The formation of liposomes is achieved by the encapsulation of the drug within a lipid bilayer, which is composed of phospholipids and cholesterol. A review of the literature indicated that the formulation of curcumin into liposomes can improve its dissolution rate, bioavailability, and stability (You et al., 2014; Laomeephol et al., 2020; Wang et al., 2021; Xi et al., 2022; Huang et al., 2023). The choice of phospholipids used to prepare the liposomes can affect their storage stability and *in vivo* bioavailability. For example, curcumin liposomes prepared using milk phospholipids showed better stability under adverse storage conditions (alkaline, oxygen, high temperature, and high humidity) compared to those prepared using krill phospholipids. However, the bioavailability of curcumin-loaded milk phospholipid liposomes was lower than those prepared using krill phospholipids (Wu et al., 2020). Compared to soy phospholipid liposomes, milk fat globule membrane (MFGM) liposomes exhibited higher encapsulation efficiency, smaller particle size, higher absolute zeta potential, and slower *in vitro* release. MFGM liposomes also showed higher stability under conditions of Fe^3+^, light, temperature, oxygen, and relative humidity compared to soy phospholipid liposomes (Jin et al., 2016). Although liposomal formulations have demonstrated improvements in various aspects, the results have not fully met expectations. Consequently, researchers have initiated investigations into the potential of coating the liposomes with chitosan, mannitol, or carbopol to further enhance the therapeutic efficacy (Gu and Deng, 2010; Manconi et al., 2017; Li et al., 2018; Li et al., 2020; Xu et al., 2021; Zhou et al., 2021). The details are summarized in Table 4.

**TABLE 4:** Curcumin liposomes with different excipients.

Cur-liposomes	Result	References
Phospholipid, cholesterol, TPP-PEG-PLGA formulation	The relative bioavailability was 400%, and the half-life was extended by more than 3 times	Xi et al. (2022)
Prepared using soybean phospholipid and PEG10-DSPE	The formulation had a sustained-release effect With or without the addition of PEG10-DSPE, the relative bioavailability was 1322% and 466%, respectively	You et al. (2014)
Galactose-morpholine modified curcumin liposomes	This enhanced the anti-tumor efficacy of curcumin and had good *in vivo* biocompatibility	Wang et al. (2021)
Dipalmitoylphosphatidylglycerol (DMPG) liposomes	The formulation was used to induce rapid gelation of silk fibroin (SF) and delivered the anti-cancer drug curcumin, which could improve the stability of curcumin and eliminate residual cancer cells after tumor resection	Laomeephol et al. (2020)
Curcumin liposomes (Cur-LPs) for intra-articular injection	Cur-LPs inhibited the pro-inflammatory polarization of macrophages induced by LPS, reducing the expression and secretion of inflammatory factors, alleviating the infiltration of inflammatory cells and fibrosis in the subcutaneous tissue. The anti-inflammatory effects of Cur-LPs were more effective both *in vitro* and *in vivo*	Huang et al. (2023)
Liposomes coated with chitosan or hyaluronic acid	Curcumin, with improved biocompatibility, protected A549 cells from hydrogen peroxide-induced oxidative stress. The synergistic effect of curcumin and hyaluronic acid further enhanced the proliferative effect and subsequent relative metabolic activity of the cells up to 120%	Manconi et al. (2017)
Chitosan-coated liposomes prepared using phospholipids/phosphatides and cholesterol	The relative bioavailability of the uncoated, coated, and phospholipid liposomes was 160%, 1184%, and 846.5%, respectively. The cumulative release rates of the formulations prepared with lecithin and phospholipids in HCl were 1.81 and 1.8 times higher than the pure drug, respectively, and in PBS, they were 1.97 and 2.6 times higher, respectively	Li et al. (2018), Li et al. (2020)
Chitosan-coated liposomes prepared using rhamnolipids and phospholipids	The bioavailability of curcumin in the chitosan-coated Cur-RL-Lips was higher than that in Cur-RL-Lips, but the curcumin release rate of Cur-RL-Lips was higher than the chitosan-coated Cur-RL-Lips	Zhou et al. (2021)
Mannitol-coated liposomes prepared using cholesterol and soybean phospholipids	The Cmax was more than 4 times that of the pure drug, and the relative bioavailability was 3189%	Xu et al. (2021)
0.5% Carbopol-coated liposomes prepared using soy phospholipids and cholesterol	The relative bioavailability of the uncoated and coated liposomes was 127% and 281%, respectively. The uncoated liposomes had a 24-h cumulative release of 53.5% in a mixed solution of PBS and ethanol at a volume ratio of 1:25, which was 9.7 times that of the pure drug	Gu and Deng (2010)

Although curcumin liposomes possess the potential to enhance therapeutic efficacy, their development is hindered by several limitations. Firstly, conventional techniques for liposome preparation, such as the thin-film hydration method and ethanol injection, often result in challenges such as low encapsulation efficiency, inconsistent particle size, and the presence of residual organic solvents. Therefore, it is difficult to achieve industrial production. Secondly, the excipients utilized in targeted liposomes, including galactose-modified curcumin liposomes, frequently lack established standards for production and quality control, making it difficult to ensure their safety and efficacy. Lastly, the excipients employed in these liposomes may provoke adverse effects, including immune responses and allergic reactions, as a result of their insolubility and non-biodegradability. Therefore, curcumin liposomes are still only at the laboratory stage at present, and a large amount of basic research is still needed in the future.

### 4.7 Microspheres and microcapsules

Curcumin-loaded microspheres have been developed for inflammatory treatment. For example, gelatin/silk fibroin (30/70) microspheres encapsulating curcumin have been demonstrated to delay the destruction of joint and synovial tissues, rendering them useful for the local treatment of osteoarthritis (Ratanavaraporn et al., 2017). Curcumin-conjugated chitosan microspheres also exhibit good antimicrobial, antioxidant, and anti-inflammatory activities, which can prevent skin infections (Saranya et al., 2018). Furthermore, curcumin microspheres are often coated with Eudragit^®^ S100 to achieve colon-specific delivery for the treatment of colon cancer. Karade et al. developed Eudragit^®^ S100-coated curcumin microspheres containing ascorbic acid (AA), which improved the stability of curcumin under alkaline conditions and prevented its degradation (Karade and Jadhav, 2018). Zhang et al. prepared Eudragit^®^ S100-coated calcium alginate curcumin microspheres, which showed significantly increased curcumin release in simulated colonic fluid (Zhang L. et al., 2011). The microencapsulation of curcumin can also improve its stability and cell compatibility, thereby enhancing its therapeutic efficacy. For instance, the lotus seed protein-pectin composite microcapsules designed for curcumin significantly enhance its antioxidant properties, photostability, thermal stability, and storage longevity (Su et al., 2022). Research demonstrates that the microencapsulated curcumin provides a sustained, controlled release during the gastric phase, which transitions to a rapid release in the intestinal phase. Following gastrointestinal digestion, the cumulative release rates for curcumin-LSP and curcumin-LSP-pectin particles are found to be 64.3% and 72.4%, respectively. Microspheres and microcapsules can encapsulate drugs within excipients, enabling the modulation of drug release rates for sustained and controlled release. This technology holds significant promise for widespread clinical applications.

### 4.8 Microemulsions

The typical composition of curcumin microemulsions includes an oil phase (e.g., ethyl oleate), an emulsifier (e.g., polyoxyethylene 40 hydrogenated castor oil), and a co-emulsifier (e.g., polyethylene glycol 400). Zhong et al. developed a liver-targeted curcumin microemulsion by adding N-octadecyllactobionamide as a liver-targeting ligand, achieving a high drug loading of up to 95% (Zhong et al., 2019). Stability experiments have demonstrated that drug-loaded microemulsions exhibit remarkable stability across a range of concentrations, storage times, high-speed centrifugation, and varying K_m_ conditions. This underscores the potential benefits of microemulsions as carriers for controlled-release and targeted drug delivery. In this context, Liu utilized dioctyl sulfosuccinate as an effective charge modifier to formulate a curcumin-targeted self-microemulsion intended for inflammatory conditions (Liu, 2017). By modifying the surface charge of the particles, the negatively charged curcumin self-microemulsion system achieved a high encapsulation efficiency and exhibited a uniform particle size distribution. This design enabled precise targeting of curcumin to the sites of inflammation in colitis, thereby increasing the concentration of the drug on the surface of inflammatory cells in ulcerative colitis. Zhang added a folic acid-polyethylene glycol-cholesterol hemisuccinate ester together with Cremophor EL, which enhanced the absorption of curcumin in the rat colonic mucosa (Zhang, 2012). Furthermore, microemulsions care capable of penetrating the stratum corneum, rendering an optimal choice for topical drug delivery (Luna-Canales et al., 2023). A redox-responsive microemulsion based on the surfactant selenium diol (PSeP) has been demonstrated to significantly improve the solubility, stability, antioxidant capacity, and skin permeability of curcumin in the oil phase, rendering it a promising system for topical applications (Zhang et al., 2023). Therefore, curcumin microemulsion shows considerable advantages in the treatment of skin diseases, promising potential for entry into clinical trials as a novel therapeutic agent for transdermal delivery.

### 4.9 Microtablets

Curcumin microtablets can be classified into three principal categories: membrane-controlled microtablets, matrix-type microtablets, and matrix-membrane-controlled sustained-release microtablets. Jv et al. prepared membrane-controlled microtablets using chitosan and sodium alginate as the core materials, and then coated them with Eudragit S100 to effectively control the release of curcumin from a self-microemulsion and achieved colon-targeted sustained drug delivery (Jv et al., 2022). Matrix-type microtablets are frequently prepared using microcrystalline cellulose as the matrix material and glyceryl monostearate as the binder. Wang additionally incorporated mucoadhesive materials, such as chitosan and hydroxypropyl methylcellulose K100M (HPMC K100M), into the formulation of gastro-retentive microtablets (Wang, 2019). This prolonged the gastric residence time and increased the contact area between curcumin and the gastric mucosa, thereby enhancing absorption and improving therapeutic efficacy. Wang incorporated HPMC K100M and the effervescent agent sodium bicarbonate to prepare curcumin gastro-floating microtablets (Wang, 2017). These exhibited sustained-release properties and remained buoyant in simulated gastric fluid for more than 8 h. In comparison to traditional curcumin tablets, the gastro-floating microtablets of curcumin demonstrate a remarkable increase in relative bioavailability, achieving a value of 271.23%. Zhao et al. developed matrix-membrane-controlled sustained-release curcumin microtablets using ethylcellulose and microcrystalline cellulose as the matrix materials, carbopol as the retardant, and stearic acid as the binder (Zhao et al., 2015). The microtablets were subsequently coated with a Kollicoat SR30D dispersion containing talc and PEG6000. The sustained-release microtablets demonstrated a drug release of 90.54% within 12 h, in accordance with the Weibull equation. The incorporation of these excipients extends the duration of drug release, improves the medication’s stability and bioavailability, and ultimately facilitates a reduction in both the required dosage and the frequency of administration. Currently, the technology for microtablets is quite mature. Curcumin microtablets can significantly enhance the dissolution rate of curcumin, thereby improving its therapeutic efficacy. Consequently, curcumin microtablets hold promise for entry into clinical trials, poised to become a novel medication for disease treatment.

### 4.10 Pellets

Curcumin pellets can be classified into immediate-release pellets and sustained-release pellets.Immediate-release pellets are frequently prepared using water-soluble matrices such as polyethylene glycol (PEG). In contrast, sustained-release pellets are typically formulated using lipophilic matrices, including stearic acid and surfactants, such as poloxamer. PEG is a frequently utilised matrix for immediate-release pellets, and the molecular weight of PEG can affect the performance. Wang et al. found that using a lower viscosity PEG4000 resulted in better pellet formation and appearance compared to the higher viscosity PEG6000 (Wang et al., 2013). Hong et al. and Chen et al. used a combination of PEG1000 and PEG6000 (Hong et al., 2013), or PEG4000 and PEG6000 (Chen et al., 2007), respectively, and optimized the formulation by investigating factors, such as drug solution temperature, cooling medium temperature, and matrix ratio to achieve desirable appearance and dissolution characteristics. Fang et al. employed lipophilic matrices, such as glyceryl monostearate and stearic acid, in conjunction with various surfactants, including polyethylene glycol-12-hydroxystearate (Solutol), polyoxyethylene hydrogenated castor oil, and poloxamer, to create sustained-release pellets (Fang et al., 2010). The results indicated that the pellets containing Solutol exhibited significantly better *in vitro* release performance compared to those with poloxamer or polyoxyethylene hydrogenated castor oil. Research on curcumin pellets has largely stagnated since initial studies conducted over a decade ago, with no recent developments reported in this area. Additionally, the existing pellets have not been subjected to efficacy assessments. As a result, based on available data, progressing curcumin pellets to Phase I clinical trials presents significant challenges.

### 4.11 Cyclodextrin inclusion complexes

The preparation of curcumin/cyclodextrin polymer (CUR/CDP) inclusion complexes has the potential to improve the solubility and stability of curcumin (Chen et al., 2020). These inclusion complexes have been demonstrated to exhibit enhanced cytotoxicity and apoptotic effects on cancer cells (Roozbehi et al., 2020). It has been demonstrated that CUR/CDP exhibits novel antioxidant activities by scavenging 2,2′-azino-bis(3-ethylbenzothiazoline-6-sulfonic acid) (ABTS) and 1,1-diphenyl-2-picrylhydrazyl (DPPH) free radicals. Furthermore, it has been shown to demonstrates enhanced antiproliferative activity and to induce apoptosis in human malignant melanoma (A375) cells (Chen et al., 2018). Furthermore, CUR/CDP has been demonstrated to enhance the activity of the antioxidant enzyme catalase (CAT), reduce lipid peroxidation, and alleviate oxidative stress, thereby inhibiting cell apoptosis and mitigating liver injury (Chen J. et al., 2022). The incorporation of cyclodextrin inclusion complexes into composite membranes has been demonstrated to enhance the therapeutic efficacy of antimicrobial membranes based on 2,3-dialdehyde cellulose (DAC) or cationic starch and polyvinyl alcohol polymer membranes (MBN). The DAC-composite film exhibits high transparency, yet effectively blocks UV light, which helps delay the degradation of curcumin (Chen et al., 2021). The CUR/CDP incorporated with MBN can specifically bind to target cancer cells, enhancing the cytotoxicity of curcumin against both melanoma and glioblastoma cell lines, and prolonging the cytotoxic effect up to 96 h (Gularte et al., 2020). Cyclodextrin inclusion significantly improves the stability, solubility, and bioavailability of curcumin. Additionally, being a naturally occurring cyclic oligosaccharide, cyclodextrin is notable for its biocompatibility and biodegradability. As a result, complexes formed between curcumin and cyclodextrin demonstrate considerable promise for various clinical applications.

### 4.12 Gel formulations

Curcumin gel formulations are typically composed of curcumin and an aqueous gel matrix, with the addition of moisturizers, pH adjusters, and permeation enhancers as needed. Liu et al. prepared curcumin gels by incorporating moisturizers and pH adjusters, resulting in good gel formation, spreadability, and thermal and cold resistance (Liu X. Y. et al., 2024). Zhu et al. incorporated gglycyrrhizic acid and polyoxyethylene castor oil (Cremophor EL) into the curcumin gel formulation as permeation enhancers (Zhu et al., 2021). The findings demonstrated that the incorporation of permeation enhancers markedly enhanced the dermal permeation of curcumin, with glycyrrhizic acid exhibiting superior efficacy compared to Cremophor EL at equivalent concentrations. Additionally, gels possess adhesive properties, enabling them to adhere to nasal mucosa, cornea, and conjunctiva, thereby prolonging the drug’s residence time. Chen et al. developed an *in-situ* nasal gel using poloxamer 407, poloxamer 188 as the gel matrix, and PEG400, 0.02% benzalkonium chloride, and 0.9% sodium chloride (Chen et al., 2013). This gel not only extended the pharmacological effect of curcumin but also significantly enhanced its brain targeting properties. Li et al. prepared a curcumin nano-suspension gel that improved the dissolution of curcumin (Li et al., 2017). *In vivo* studies demonstrated that this gel formulation could significantly delay the development of lens opacification and improve the therapeutic effect of curcumin on selenium-induced cataracts in rats. Currently, curcumin gel formulations are undergoing phase III clinical trials for the treatment of periodontitis. This suggests that curcumin gel may have the potential to become a new therapeutic option for dental diseases.

### 4.13 Film and coating formulations

Curcumin film and coating formulations are commonly used for food preservation and wound healing. Han et al. prepared curcumin films using polyvinyl alcohol (PVA 17-88) and deacetylated chitosan as film-forming materials, with glycerol as a plasticizer (Han et al., 2005). The resulting films exhibited favourable film-forming properties and could promote wound healing. Compared to films, coating formulations are more convenient for topical application, as the protective film forms automatically after spraying. The use of permeation enhancers [such as menthol (Chen J. H. et al., 2022) and dimethyl sulfoxide (Chen, 2015)], facilitates the continuous penetration of the active compounds in the film through the skin, thereby ensuring sustained absorption of curcumin. Furthermore, the incorporation of chitosan and its derivatives in the coating formulations can enhance the binding of curcumin to bacteria, thereby improving the antimicrobial activity (Zhang et al., 2024). Cao et al. developed a composite film containing carboxymethyl chitosan, carboxymethyl cellulose, and curcumin, which effectively inhibited the growth of Aspergillus niger and Penicillium, and had a protective effect on food color, making it widely applicable for food preservation (Cao et al., 2022). Thus, curcumin films and coatings demonstrate significant antibacterial properties, offering promising potential for innovative applications in food preservation and wound healing.

### 4.14 Vesicles

Curcumin vesicles are typically composed of surfactants and cholesterol, with common non-ionic surfactants being Span and Tween. Wang prepared curcumin vesicles by dissolving Span 80, cholesterol, and curcumin in ethanol, evaporating the solvent, and then hydrating the mixture with pH 6.5 PBS (Wang, 2017). The results indicated that the bioavailability of curcumin was significantly improved compared to a curcumin suspension. Lv formulated curcumin vesicles by dissolving Span 60, cholesterol, and curcumin in a chloroform: ethanol (4:1) solution, evaporating the solvent, and then hydrating with ultrapure water (Lv, 2022). After transferrin modification, these vesicles could effectively target and treat glioblastoma, with good safety profiles *in vivo*. Hao et al. prepared curcumin vesicles using Tween 60 and Tween 80, and found that the Tween-60 vesicles were more effective in improving the UV absorption, fluorescence emission intensity, binding constant, and free radical scavenging ability of curcumin compared to the Tween-80 vesicles (Hao et al., 2021). While curcumin vesicles have been shown to improve the effectiveness of curcumin, the existing research on their distribution, metabolism, and clearance within the body is still limited. Additionally, the long-term safety of these vesicles has yet to be established, which is why they are not yet suitable for advancement to clinical trial phases.

### 4.15 Tablet formulations

Tablets represent a traditional and common dosage form, with relatively low production costs and simple manufacturing processes. Zheng et al. prepared curcumin sustained-release tablets using a direct compression method with a single-punch tablet press (Zheng et al., 2015). They used sodium dodecyl sulfate as a solubilizer, anhydrous lactose as a pore-forming agent, talc and magnesium stearate as lubricants, and corn starch as a filler. Carbopol and HPMC K4M were used in combination as the sustained-release materials, resulting in an 80% drug release in 8 h. Zhao et al. combined phospholipid complexation and PVP K30 solid dispersion techniques to prepare a compound curcumin solid dispersion tablet (Zhao et al., 2014). This tablet formulation could promote drug disintegration, increase gastrointestinal absorption, slow down the elimination rate, and inhibit the distribution, thereby improving the bioavailability of curcumin. Furthermore, tablets can be combined with various innovative technologies, including solid dispersion and cyclodextrin inclusion. This synergy not only improves the dissolution and bioavailability of curcumin but also enhances portability and ease of use. As such, these formulations are poised to become the favored choice in clinical trials.

## 5 Clinical research on curcumin

As the solubility and bioavailability of curcumin have improved, clinical research on curcumin has begun to gain momentum. The majority of existing clinical studies are primarily focused on Phase I and Phase II (Figure 1C). Phase I clinical trials are primarily concerned with the assessment of the drug’s safety and tolerability. Dose escalation studies have demonstrated that oral doses of curcumin as high as 12 g/d do not result in significant toxicity in the short term (Liu et al., 2013). Furthermore, numerous trials have demonstrated the safety and efficacy of curcumin in the treatment of diabetes, osteoarthritis, cancer, and inflammatory diseases. A survey conducted up to 2023 revealed that the majority of curcumin clinical trials, 22%, are related to metabolic abnormalities associated with obesity and insulin resistance. This is followed by musculoskeletal diseases (MSK, 17%), neurological diseases (NEURO, 11%), gastrointestinal diseases (GI, excluding non-alcoholic fatty liver, 10%), and cancer (CA, 9%) (Panknin et al., 2023). For further details, please refer to Table 5. In a clinical trial for T2DM, Adibian M et al. administered 1,500 mg of curcumin daily for 10 consecutive weeks (Adibian et al., 2019). The results demonstrated that curcumin reduced total cholesterol levels and inflammatory markers; In a study conducted by Hodaei et al., a dosage of the same medication was found to result in a reduction in fasting blood sugar and body weight in patients (Hodaei et al., 2019). The addition of a small amount of piperine (PIP) to curcumin has been demonstrated to lower the atherogenic lipid index in the serum, as well as to improve blood sugar and liver parameters in patients with T2DM (Panahi et al., 2017; Panahi et al., 2018). A daily supplementation of 70 mg of curcumin for 8 weeks has been demonstrated to improve liver fat content and body mass index in patients with non-alcoholic fatty liver disease (Rahmani et al., 2016). Several clinical studies have demonstrated that curcumin exhibits significant therapeutic efficacy against major metabolic complications associated with the development and progression of metabolic disorders, including obesity, T2DM, and non-alcoholic fatty liver disease. These findings highlight the potent anti-inflammatory and antioxidant properties of curcumin.

**TABLE 5:** Clinical trials of curcumin.

Disease	Dose	Duration	Results	Molecular mechanisms	References
T2DM	1500 mg/d	10 w	Lowered total cholesterol levels and inflammatory markers	TNF-α, IL6 ↓ NF-κB ↑	Adibian et al. (2019)
	1500 mg/d	10 w	Reduced fasting blood sugar and body weight in patients	PPAR activity ↑	Howells et al. (2019)
	1000 mg/d, pip 10 mg/d	12 w	Lowered the level of atherosclerotic lipid index in the serum	—	Panahi et al. (2017)
	500 mg/d, pip 5 mg/d	3 m	Improved blood sugar and liver parameters in patients	NF-κB ↑ cyclooxygenase-2, ICAM-1, MCP-1, and nitric oxide production ↓	Panahi et al. (2018)
Overweight and obesity	500 mg/d	10 w	Beneficial effects on body mass index, waist circumference, hip circumference, high-density lipoprotein levels, and triglyceride/high-density lipoprotein ratio	fatty acid β-oxidation and lipoprotein lipase ↑, fatty acid synthase, lipogenic genes and enzymes ↓	Saraf-Bank et al. (2019)
Metabolic syndrome	200 mg, coenzyme Q10 60 mg/d	12 w	Improved lipid abnormalities	HMG-CoA reductase, SREBPs and fatty acid synthase ↓	Sangouni et al. (2022)
Non-alcoholic fatty liver disease	70 mg/d	8 w	Significantly reduced liver fat content, body mass index, serum total cholesterol, low-density lipoprotein cholesterol, triglycerides, alanine aminotransferase, aspartate aminotransferase, glucose, and glycated hemoglobin levels	NF-κB ↑, oxidative stress ↓	Rahmani et al. (2016)
Osteoarthritis	350 mg, boswellic acid 150 mg × 3/d	12 w	Alleviated pain-related symptoms in OA patients	IL-1β, TNF-α ↓	Haroyan et al. (2018)
	80 mg/d	3 m	Significant reductions in visual analog scale (VAS), C-reactive protein (CRP), CD4 and CD8 T cells, Th17 cells, and B cell frequencies	IL-1β, IL-17 ↓	Atabaki et al. (2020)
	80 mg × 2/d	90 d	Effectively and safely relieved symptoms in patients with knee osteoarthritis	IL-1β, TNF-α ↓	Gupte et al. (2019)
Rheumatoid arthritis	40 mg × 3/d	12 w	No significant impacted on disease activity score (DAS-28), tender joint count (TJC), and swollen joint count (SJC)	—	Javadi et al. (2019)
Migraine	80 mg/d	2 m	Significantly reduced gene expression and plasma levels of IL-17	IL-6, hs-CRP serum levels, and TNF-α ↓	Djalali et al. (2020)
Severe depression	80 mg × 2/d	8 w	Marked improvements in total score and emotional score of IDS-SR30	CRP, IL-6, and TNF-α ↓	Lopresti et al. (2014)
Gastrointestinal barrier damage	500 mg/d	3 d	Improved gastrointestinal function, related cytokines, and systemic physiological responses during exertional heat stress	IL-1RA, IL-6, IL-10, MCP-1, and TNF-α ↓	Szymanski et al. (2018)
*Helicobacter pylori* gastric infection	700 mg × 3/d	8 w	Reduced markers of malondialdehyde, glutathione peroxidase, and increased total antioxidant capacity of the gastric mucosa	TAC, NF-κB ↑, GPx, MDA ↓	Judaki et al. (2017)
Head and neck cancer	4000 mg/d	8 w	Significantly increased muscle mass, improved and delayed other body composition parameters, grip strength, and decreased absolute lymphocyte count	NF-κB ↑	Thambamroong et al. (2022)
Colorectal cancer	480 mg, quercetin 20 mg × 3/d	6 m	Reduced the number and size of adenomas in the colon and rectum in patients, with no apparent toxicity	glutathione S-transferases ↑ isoenzyme cyclooxygenase-2 ↓	Cruz-Correa et al. (2006)
Polycystic ovary syndrome	500 mg × 3/d	12 w	Improved hyperandrogenism and hyperglycemia related to PCOS	FSH, LH, IL-1β, and TNF-α ↓, PGC-1α ↑	Heshmati et al. (2021)
Chronic obstructive pulmonary disease	90 mg × 2/d	24 w	Lowered levels of atherosclerotic AT-LDL	TNF-α, COX-2, STAT, cyclin D1, and NF-κB ↓	Funamoto et al. (2016)

Osteoarthritis is the most prevalent musculoskeletal disease treated with curcumin, with Atabaki M et al. found that curcumin can reduce Visual Analog Scale (VAS) scores, C-reactive protein (CRP), CD4 and CD8 T cells, Th17 cells, and B cell frequencies to alleviate osteoarthritis (Atabaki et al., 2020). However, the therapeutic effects of curcumin on rheumatoid arthritis, which is also classified as a musculoskeletal disorder, have not demonstrated significant efficacy (Javadi et al., 2019). The addition of boswellic acid can reduce pain-related symptoms in patients (Haroyan et al., 2018). Furthermore, curcumin has demonstrated efficacy in the treatment of various neurological disorders, including migraines and severe depression. A daily intake of 80 mg of curcumin resulted in a significant reduction in the gene expression and plasma levels of IL-17 in migraine patients after 2 months, leading to an improvement in migraines (Djalali et al., 2020); while a daily intake of 160 mg of curcumin was found to result in a discernible improvement in mood after 8 weeks (Lopresti et al., 2014). Clinical trials indicate that curcumin, due to its exceptional antioxidant and anti-inflammatory properties, can ameliorate the symptoms of gastrointestinal barrier damage during *Helicobacter pylori* gastric infection and exertional heat stress (Judaki et al., 2017; Szymanski et al., 2018). Furthermore, clinical trials have indicated that curcumin, when used in combination with other drugs or treatments, can enhance treatment efficacy and reduce the side effects of other drugs. The combination of curcumin, high taurine (100 μM) and vitamin D3 (50 nM) is employed in the treatment of diabetic retinopathy (Filippelli et al., 2020). Studies have demonstrated that while each of the agents in question has a relatively weak anti-inflammatory effect when used in isolation, their combined use can result in a significant alteration of the average levels of soluble mediators of inflammation and retinal damage. In a series of clinical trials, Howells LM and colleagues demonstrated that curcumin is a safe and tolerable adjunctive medication for FOLFOX chemotherapy in patients with metastatic colorectal cancer (Howells et al., 2019).

In recent years, there has been a notable increase in the number of clinical registrations for curcumin at Phase III and Phase IV. India has emerged as the primary country responsible for these registrations. These registrations are mainly focused on oral and dental diseases, arthritis, and diabetes (Table 6). The primary research subjects within the field of oral and dental diseases are oral mucositis and periodontitis. India has consecutively registered clinical trials related to oral mucositis in 2017 (CTRI/2017/01/007732, CTRI/2017/08/009461, CTRI/2017/09/009674), 2020 (CTRI/2020/10/028666), 2021 (NCT04896164, CTRI/2021/01/030470), 2022 (CTRI/2022/08/044967), and 2023 (CTRI/2023/07/055584). Additionally, nearly half of these trials originate from the Maulana Azad Institute of Dental Sciences, which indirectly indicates the potential therapeutic effects of curcumin in clinical treatment of this disease. However, further extensive clinical verification and refinement are still required to establish a solid data foundation for clinical application. In 2017, India registered a clinical trial for the use of daily curcumin tablets at 500 mg for a continuous 6-week period (CTRI/2017/11/010494), followed by the registration of a clinical trial in 2019 for the application of curcumin gel for a continuous 1-year period (CTRI/2019/04/018697). Furthermore, in 2022, a clinical trial for the use of curcumin-containing toothpaste for a continuous 7-month period (CTRI/2022/07/043703) to treat periodontitis was also registered. In July 2019, the University of Ains Sham registered a clinical trial for the adjunctive treatment of curcumin paste (NCT04032132), followed by the registration in August of the same year for a clinical trial using curcumin-simvastatin-EDTA (NCT04044417) to treat periodontitis. The employment of diverse dosage forms and administration methods in Phase IV clinical trials will permit a multidimensional exploration of the actual effects of curcumin on periodontitis in clinical practice. This may facilitate the earlier application of the findings in clinical settings. In summary, curcumin represents a promising novel therapeutic agent for the treatment of oral and dental diseases. Since 2018, there has been a notable increase in the number of clinical trials for musculoskeletal diseases such as arthritis and rheumatoid arthritis that have reached Phase III and Phase IV. India registered the use of curcumin-pip tablets for the treatment of rheumatoid arthritis and early knee osteoarthritis in 2018 (CTRI/2018/04/013279) and 2022 (CTRI/2022/07/043621) respectively. Following a 4-year interval, the same medication was registered for the treatment of different types of arthriti. This led us to speculate that curcumin-pip tablets may have certain clinical efficacy for rheumatoid arthritis. Furthermore, experts have successively conducted clinical trials on diabetes and its complications in multiple countries including Thailand (NCT01052025), Egypt (NCT04528212), Mexico (NCT03262363, NCT03917784), and Iran (NCT02529969) to explore the therapeutic effects of curcumin on diabetes. Over the past 5 years, there have been only two Phase III and Phase IV clinical trials related to cancer treatment (CTRI/2023/02/050028, CTRI/2019/03/018265). A review of clinical trials conducted between 2005 and 2015 revealed a number of studies related to colorectal cancer and pancreatic cancer in Israel (NCT00295035, NCT00486460), prostate cancer in the United States (NCT02064673), and In India, clinical trials related to squamous cell head and neck cancer and advanced cervical cancer (CTRI/2008/091/000195, CTRI/2008/091/000131) have yet to yield clear clinical outcomes. Moreover, no favourable clinical results have been reported. This leads us to hypothesize that the role of curcumin in clinical cancer treatment is minimal or non-existent.

**TABLE 6:** Curcumin phase III and Phase IV clinical registration.

Identifier	Drug name	Contractor	Disease	Phase
NCT05852366	curcumin based Jasmate toothpaste, tooth brushing	Dr. D. Y. Patil Dental College and Hospital	Dentinal Hypersensitivity, Chronic Periodontitis	Phase III
NCT05627843	curcumin	Ain Shams University	Pediatric Patients Having Renal Failure and on Regular Hemodialysis	Phase III
NCT04971382	ridge splitting surgery, curcumin plus xenograft	Ain Shams University	Horizontal Alveolar Bone Loss	Phase IV
NCT04896164	curcumin	Terry Fox Research Institute	Oral Mucositis (Ulcerative)	Phase III
NCT04837664	Curcumin, Rose Bengal, Nystatin	King Saud University	*Candida* albicans infection, Cigarette Smoking	Phase III
NCT04528212	glimepiride, Variable regimens including black pepper, curcumin, fenofibrate, glimepiride	Damanhour University	Diabetes Mellitus, Type 2	Phase IV
NCT04205929	curcumin	Oregon Health & Science University	Bleeding, Implants, Breakthrough Bleeding	Phase IV
NCT04044417	currettage, curcumin simvastatin paste, EDTA root conditioning	Ain Shams University	Periodontitis	Phase IV
NCT04032132	curcumin paste, curettage	Ain Shams University	Periodontitis	Phase IV
NCT03917784	Curcumin, Starch	Hospital General de México Dr. Eduardo Liceaga	PreDiabetes	Phase IV
NCT03790605	1% curcumin chip, Placebo chip, Scaling and root planing	KLE Society’s Institute of Dental Sciences	Periodontitis	Phase III
CTRI/2023/10/059007	Aloe Vera paste, Aloe Vera paste, Curcumin gel, Curcumin gel	Dr Pavithra M	T2DM without complications	Phase IV
CTRI/2023/07/055584	Curcumin, Lycopene	Sardar Patel Post Graduate Institute of Dental and Medical Sciences	Oral submucous fibrosis	Phase III
CTRI/2023/02/050028	Curcumin, Placebo	—	Malignant neoplasm	Phase III
CTRI/2022/11/047253	Selective deep caries excavation, Two dyes are used (Curcumin and Indocyanine green)	Dr Sonali Khandelwal	Dental caries, unspecified	Phase III
CTRI/2022/09/045864	Group A (Experimental sites) intrasulcular application of commercially available Curcumin gel (C. Longa extract 10 mg), Group B (Control sites) simulated treatment lacking the curcumin gel	Pramod Kumar	Chronic periodontitis	Phase III
CTRI/2022/08/044967	Topical Curcumin Gel (1%), Topical Pirfenidone gel (8%), Topical Steroid – Triamcinalone acetonide (0.1%)	Maulana Azad Institute of Dental Sciences	Oral submucous fibrosis	Phase III/IV
CTRI/2022/07/043703	Group 1 - curcumin containing toothpaste. Group 2 - Non curcumin containing toothpaste	ARUMUGAM P M	Chronic periodontitis	Phase IV
CTRI/2022/07/043621	Curcumin-Piperine, Probiotics, Placebo for curcumin-piperine and usual care	University Grants Commission UGC NET JRFSRF Grant	Bilateral primary osteoarthritis of knee	Phase III
CTRI/2022/03/041339	1% CURCUMIN GEL, tetracycline fiber (Periodontal plus AB)	Dr Lekshmi R	Chronic periodontitis	Phase III
CTRI/2022/01/039537	BIOENHANCED CURCUMIN (CURCUMIN WITH ESSENTIAL OIL OF CURCUMIN), PLACEBO	rp centre of ophthalmic sciences	Disorders of lacrimal system	Phase III/IV
CTRI/2021/08/035401	Group A Systemic and topical curcumin, Group B Systemic and Topical curcumin	NIMS Dental College and Hospital	Oral submucous fibrosis	Phase IV
CTRI/2021/05/033886	Curcumin gel coated on gel foam, Plain gel foam (placebo)	Sri Ramachandra Institute of Higher Education and Research	Dental caries, unspecified	Phase III
CTRI/2021/02/031169	2% Curcumin gel, scaling and root planing	Grace Mary Joseph	Chronic periodontitis, T2DM without complications	Phase III/IV
CTRI/2021/01/030470	Silymarin 1% Curcumin 10 mg, Triamcinolone acetonide 0.1%	—	Oral submucous fibrosis	Phase III
CTRI/2020/10/028666	Aloe Vera gel, Curcumin Longa (Liquid), Spirulina, Triamcinolone acetonide	Pravara Institute of Medical Sciences Deemed University	Oral submucous fibrosis	Phase III/IV
CTRI/2020/07/026820	Curcumin, Placebo	Rohit Walia	people at risk of COVID infection - healthcare workers, diabetes, cardiac patients	Phase IV
CTRI/2020/03/024221	Liposomal Curcumin, NA	SSV Phytopharmaceuticals	Osteoarthritis of knee, unspecified	Phase III
CTRI/2019/11/021905	chlorhexidine mouthwash, curcumin mouthwash, curcumin rosemary mouthwash, mouthwashes	Sujitha	patients requiring fixed orthodontic treatment	Phase IV
CTRI/2019/06/019908	Group 1 Endoplasm-FS, Group 2 Endoplasm powder with Curcumin gel, Group 3 Pulpdent Root Canal Sealer Zinc oxide eugenol	Pragyna Priyadarshini	Placement	Phase III
CTRI/2019/06/019636	Bio active Liquid Curcumin, Conventional Pharmaceutical Treatment for 3 months treatment period	Stabicon Life Sciences Pvt Ltd.	T2DM without complications	Phase III
CTRI/2019/04/018697	Boric acid gel, Curcumin gel	—	Administration	Phase IV
CTRI/2019/03/018265	Bio active Liquid Curcumin, Conventional Pharmaceutical Treatment for 3 months treatment period	Stabicon Life Sciences Pvt Ltd.	T2DM without complications	Phase III

## 6 Conclusion

This article provides a comprehensive review of the research advancements related to curcumin, focusing on three key areas: pharmacological effects, formulation studies, and clinical applications. Previous reviews of curcumin have predominantly focused on specific diseases or formulations, with few examining its overall pharmacological effects, formulation research, and clinical applications comprehensively, such as Hao et al. (2023). Firstly, this review presented a comprehensive overview of the pharmacological mechanisms of curcumin. However, they fell short in establishing a link between these mechanisms and clinical outcomes. Secondly, this review highlighted poor bioavailability as a fundamental obstacle to clinical application and suggested enhancements through methodologies such as nanotechnology and solid dispersions. Nevertheless, this review did not provide more details on the challenges of implementing these formulations in clinical settings, providing only a singular example. Compared to the review by Hao et al., this article not only encapsulates the primary pharmacological effects of curcumin but also connects curcumin’s effects to clinical outcomes. This would provide more context for the potential therapeutic benefits. Furthermore, this article summarizesthe results of molecular docking of curcumin, potentially aiding further exploration of the compound’s therapeutic properties. Additionally, it underscores the progress in research on various curcumin formulations, evaluating their feasibility/infeasibility within clinical settings.

Curcumin exhibits a multitude of biological activities, including anticancer, anti-inflammatory, antioxidant, and antibacterial effects. Extensive research has been conducted to explore its mechanisms of action, establishing a theoretical foundation for future clinical trials. In addition, some researchers have utilized network pharmacology to identify curcumin’s targets across various diseases. By selecting core targets and conducting molecular docking studies, they have found that a binding energy of less than −5.0 kcal/mol indicates a strong interaction between curcumin and these targets, suggesting their potential as therapeutic candidates. Computational simulations can also be employed to predict curcumin’s therapeutic potential for different diseases, which may help mitigate the costs associated with traditional trial-and-error approaches. However, it is important to note that the accuracy of these simulations can vary due to potential biases in the underlying databases. Therefore, further animal studies and clinical observations are essential for validation.

A comprehensive review of curcumin’s pharmacological effects reveals that it possesses a diverse range of beneficial properties. When taken at a daily dosage of 12 g, curcumin shows good tolerability and does not appear to cause significant toxicity in the short term. However, its clinical application is limited by challenges such as low solubility, poor bioavailability, and a short half-life. To address these challenges, researchers both domestically and internationally have explored various formulation technologies to create innovative dosage forms, including nano-targeted formulations (such as liposomes, nanoparticles, and micelles), nanoemulsions, and nanocrystals.

Despite these advancements, current clinical trials predominantly focus on tablets or capsules. This phenomenon is worthy of our thought. Here are our views on this phenomenon. Firstly, most existing nano-targeted formulations rely on the Enhanced Permeability and Retention (EPR) effect for cancer treatment, which has demonstrated significant efficacy in animal models. However, EPR effect was not found in human (Sharifi et al., 2022). Thus, it is difficult to apply nano-targeted formulations in clinical practice. Secondly, the lack of standardized production and quality control protocols for these nano-targeted materials complicates the effective assessment of their safety and efficacy. Thirdly, excipients such as mesoporous silica, due to their insolubility, non-biodegradability, and inadequate biocompatibility, may accumulate in tissues and organs, thereby impeding excretion and potentially leading to long-term toxicity. Fourthly, the field of nano-toxicology has not kept pace with the rapid advancements in nano-formulations. In-depth studies on the toxicity mechanism of nano-materials are needed to guide the design and modification of nano-materials to reduce their toxicity and improve their biocompatibility and safety. Fifthly, although contemporary nano-targeting agents exhibit favorable physicochemical properties and pharmacological effects, the incorporation of nano-targeted materials can lead to a range of adverse effects following oral administration. For example, alterations in the composition of the human gut microbiota may contribute to obesity, metabolic syndrome, and an increased incidence of other chronic inflammatory diseases. Additionally, the stability of nano-formulations is poor, rendering them prone to aggregation, sedimentation, leakage, and structural degradation during storage and transportation, which adversely impacts their physicochemical properties and therapeutic efficacy. For example, nano-emulsions may have problems such as particle size growth, gelation, and drug precipitation during storage. In summary, nano-targeted formulations prepared under the existing conditions are difficult to meet the standards of national pharmacopoeia of various countries. Additionally, the intricate preparation processes and advanced technologies required result in significantly higher manufacturing costs compared to conventional dosage forms such as tablets and capsules. Thus, these formulations are limited to the laboratory stage. Consequently, the clinical application of nano-formulations remains challenging.

Furthermore, there are still some deficiencies in curcumin research that need to be strengthened in subsequent studies. Firstly, the drug loading capacity of the existing preparations is generally not high. The low drug loading capacity leads to insufficient drug concentration, making it difficult to achieve the expected therapeutic effect. However, to achieve the expected therapeutic effect, patients may need to increase the frequency or dosage of medication, which raise the risk of side effects. Secondly, Phase III trials are pivotal in assessing the approval of curcumin for market release, while Phase IV trials play a vital role in ongoing surveillance after approval. Together, these stages safeguard the safety, efficacy, and judicious clinical application of medications. Phase IV clinical trials not only confirm the findings of pre-market studies of curcumin but also address any biases from earlier trials, fill in gaps of missing information, and provide a solid foundation for clinical application. However, the present clinical studies in Phase III and Phase IV are limited, and the treatment of diseases is mainly focused on dental diseases, arthritis, and diabetes. A large number of cell tests and animal experiments indicate that curcumin has other therapeutic effects. However, there is a lack of substantial Phase III and Phase IV clinical evidence to demonstrate its therapeutic effects in the human body. Furthermore, the existing clinical studies mostly involve oral administration, with very few involving topical application.

In the future, polymers combination and drugs combination can be considered to enhance the efficacy of the drug and improve patient adherence through. It is also essential to develop excipients that demonstrate good biocompatibility and pose no biological toxicity, thereby minimizing side effects. Given that turmeric is a medicinal botanical drug with dietary uses, curcumin can be made into medicinal cuisine or food products to improve adherence, particularly among pediatric patients. In the future, the preparation process of curcumin should be improved, including the preparation of organic agent-free preparations and the use of biodegradable materials (polysaccharides, proteins, etc.). Furthermore, specific excipients and dosage forms can be tailored and developed based on the various types of diseases, for enhancing the rational application of formulations. For example, in treating gastric-related conditions, gastro-retentive formulations can prolong the drug’s retention in the stomach, thereby enhancing therapeutic outcomes. In conclusion, it is essential to enhance the quality control and safety assessment of pharmaceutical excipients, while conducting in-depth research on the interaction mechanisms between excipients and drugs. Furthermore, continuous innovation and development of novel pharmaceutical excipients will provide robust support for the advancement and innovation of curcumin formulations. Concurrently, the therapeutic potential of curcumin can be increased by exploring innovative targeted drug delivery strategies, improved formulation systems, and effective combination therapies. These modalities can promote its timely application in the treatment of various diseases and provide new avenues for therapeutic approaches.
